# Individual-Eye Pharmacokinetics of Intravitreal Ranibizumab: Linking Ocular Geometry to Drug Retention Using MRI and Modeling

**DOI:** 10.1167/iovs.67.12.31

**Published:** 2026-10-16

**Authors:** Patricia Lamirande, Eamonn A. Gaffney, Michael Gertz, Basil Künnecke, Thomas Bielser, Andreas Bruns, Jessica R. Crawshaw, Uwe Wessels, Antonella Rampazzo, Philip K. Maini, Antonello Caruso

**Affiliations:** 1 Wolfson Centre for Mathematical Biology, Mathematical Institute, Andrew Wiles Building, University of Oxford, Oxford, United Kingdom; 2 Department of Mathematics and Statistics and Sainte-Justine University Hospital Azrieli Research Centre, Université de Montréal, Montréal, Quebec, Canada; 3 Pharmaceutical Sciences, Roche Innovation Center Basel, Roche Pharma Research and Early Development, Basel, Switzerland; 4 Neuroscience and Rare Diseases, Roche Innovation Center Basel, Roche Pharma Research and Early Development, Basel, Switzerland; 5 School of Mathematical Sciences, Queensland University of Technology, Brisbane, Australia; 6 Pharmaceutical Sciences, Roche Innovation Center Munich, Roche Pharma Research and Early Development, Penzberg, Germany; 7 Ophthalmology Section, Vetsuisse Faculty, University of Zurich, Zurich, Switzerland

**Keywords:** magnetic resonance imaging (MRI), mathematical modeling, pharmacokinetics (PKs), intravitreal (IVT) drug delivery, ocular half-life

## Abstract

**Purpose:**

The purpose of this study was to quantify the influence of ocular geometry on intravitreal (IVT) ranibizumab pharmacokinetics (PKs) in rabbits by integrating magnetic resonance imaging (MRI)-derived geometries, diffusion-based modeling, and PK measurements.

**Methods:**

MRI was performed to obtain detailed ocular geometries in 39 New Zealand White (NZW) rabbits. Ranibizumab PKs were assessed in the same animals by serial aqueous humour (AH) sampling following single or three 0.5 mg doses. Individual eye parameters were estimated using profile likelihood analysis. A mean first passage time model was developed to predict ocular half-life as a function of eye size and injection location. Simplified ocular models parameterized by axial length (AL) were constructed to assess whether reduced anatomic representations capture population-level trends.

**Results:**

MRI-derived vitreous geometries enabled quantification of inter-eye anatomic variability. The study cohort of adult rabbits exhibited a limited range of AL and vitreous volume compared to human variability. Modeling predicted increased ocular half-life with eye size, a trend reproduced by simplified AL-based models. Experimentally estimated half-lives ranged between 2.8 and 4.9 days and showed weak correlation to AL and vitreous volume. The population mean half-life was 3.67 days, consistent with previous results.

**Conclusions:**

This study provides a quantitative framework for analyzing IVT PKs at the level of individual eyes. Modeling predicts that ocular geometry influences drug elimination, and this relationship was also detected experimentally in rabbits, albeit over a limited anatomic range. Greater variability in human eye size may therefore contribute to interindividual PK differences and warrants further investigation, as it may inform personalized dosing strategies.

Retinal vascular diseases, such as wet age-related macular degeneration (wet AMD), diabetic macular edema, and retinal vein occlusion, are major causes of vision loss worldwide.^1^^–^^3^ These disorders are characterized by pathological vascular leakage and neovascularization driven by elevated vascular endothelial growth factor (VEGF), which contributes to macular fluid accumulation and retinal damage.^1^^,^^4^ Intravitreal (IVT) injections of anti-VEGF agents, such as ranibizumab (Lucentis; Genentech/Novartis), are the standard first-line therapy to reduce abnormal vessel growth and fluid accumulation, stabilize or improve vision, and slow disease progression.^1^^,^^5^^,^^6^ However, anti-VEGF therapeutic agents have limited intraocular residence time, requiring frequent injections that impose a substantial burden on patients and healthcare systems.^7^^–^^9^ Clinical studies report substantial interindividual variability in treatment durability,^10^ with ranibizumab treat-and-extend regimens requiring between 7 and 12 injections in the first year.^11^

The mechanisms underlying this variability are not fully understood, but differences in pharmacokinetics (PKs) play a key role. Clinical PK studies report interindividual variability in IVT half-life; for example, Avery et al.^12^ estimated a mean ocular half-life of 5.8 days, with a standard deviation of 1.8 days for ranibizumab. Vitreal drug elimination is governed by both ocular and molecular size, as these factors determine diffusion and elimination dynamics within the eye.^13^^,^^14^ In previous work,^15^ we developed a diffusion-based mathematical modeling framework to examine how interindividual variation in ocular geometry influences ocular half-life, showing that variability in human eye dimensions can produce measurable differences in predicted IVT drug retention. However, experimental evidence supporting this relationship remains limited. One study of 41 eyes found no correlation between aqueous humor concentrations of ranibizumab or bevacizumab and axial length (AL).^16^ Previous studies evaluating correction for ocular volume likewise suggested minimal effects on IVT PKs.^17^^,^^18^ The present study therefore seeks to experimentally evaluate predictions generated by this mathematical modeling framework using rabbits, an established animal model for IVT PK studies that is widely used in ocular research to support translational understanding of human drug disposition.^19^^–^^23^

IVT PK studies in rabbits rarely report ocular dimensions, despite substantial differences in body weight across study cohorts. For example, rabbit body weights in previous PK studies^19^^,^^23^ range from approximately 1.5 to 2 kg to 3.1 to 4.1 kg. Reported ocular half-life estimates also differ between these studies, with mean values of approximately 2.75 and 3.3 days, respectively. Because eye size increases with age and body weight in rabbits,^24^^,^^25^ unreported anatomic variation may influence IVT drug retention. Consistent with predictions from our previous computational modeling work,^15^ such variability could contribute to discrepancies in reported PK parameters between studies.

To investigate this hypothesis, we combine mathematical modeling with new experimental measurements, using imaging to quantify individual ocular geometry and serial aqueous humour (AH) sampling to estimate IVT half-life in the same eyes.

Several techniques are available to measure eye dimensions in vivo. Magnetic resonance imaging (MRI) provides the most comprehensive anatomic assessment and has been widely applied in ophthalmic research,^26^^–^^29^ including rabbit studies.^30^^,^^31^ MRI is commonly used to characterize ocular dimensions,^28^^,^^32^^–^^35^ and the resulting data can be used to generate three-dimensional (3D) computational replicas of biological structures,^36^ including full-eye geometries of the human eye.^37^ However, MRI requires specialized equipment and expertise, limiting accessibility. In contrast, ocular biometry is more practical and affordable, and this noninvasive technique can measure axial dimensions such as anterior chamber depth, lens thickness, and AL in human eyes.^32^^,^^38^ Whereas MRI provides all possible ocular measurements and a comprehensive 3D geometry, ocular biometry is generally restricted to the measurements along the visual axis, but it is broadly accessible.

PK studies of IVT drugs are often performed by sampling the AH at different times after the injection.^14^^,^^39^^–^^41^ Drug concentrations measured in the AH samples serve as a proxy for vitreous levels, providing an indirect assessment of intraocular distribution and elimination. Our previous modeling work^15^ showed that the position of the injection within the vitreous—specifically, the location of the needle tip at the time of delivery—might influence ocular half-life. In rabbit PK studies, the IVT needle entry site is commonly described as 3 to 5 mm posterior to the anatomic limbus or, alternatively, 1 to 2 mm posterior to the surgical limbus, depending on the reference point used.^19^^–^^22^^,^^42^^–^^44^ Despite this convention, the actual position of the needle tip within the vitreous is not precisely ascertained in vivo. As a result, variability in injection location may contribute to differences in vitreal elimination, although its impact has not been experimentally quantified.

The influence of ocular geometry and injection location on IVT PK can be described using a mean first passage time (MFPT) model.^15^ In this framework, we consider isotropic diffusion and the absence of convection in the distribution of the injected ocular drug, and the MFPT is a measure of its vitreal residence time. However, these modeling predictions have not yet been experimentally validated.

The present study extends this MFPT modeling approach to examine how inter-eye variability influences ocular half-life for ranibizumab, a widely used anti-VEGF therapeutic agent and a standard antibody fragment format in ocular PK research. By combining MRI-informed MFPT modeling with individual-eye PK measurements, we test whether differences in ocular dimensions correlate with measured ocular drug half-life. This question is clinically relevant because human eyes exhibit substantial anatomic variability. If IVT PKs depend on ocular geometry, this variability may contribute to interindividual differences in treatment durability and could ultimately inform personalized dosing strategies for IVT therapies.

## Methods

### Study Design

The animal study was conducted at F. Hoffman-La Roche (Basel, Switzerland). The test facility is accredited by the Association for Assessment and Accreditation of Laboratory Animal Care International (AAALAC) and follows animal health and welfare guidelines. All procedures were in accordance with Swiss regulations and approved by the Cantonal Ethical Committee for Animal Research (studies SRS1638, SRS1836, and SRS1837).

Thirty-nine adult female New Zealand White (NZW) rabbits were obtained from a commercial supplier (Envigo UK, Inotiv, Hillcrest, UK) and acclimated under standard housing conditions for at least 7 days before study initiation. At the time of dosing, the rabbits were 23 to 27 weeks old and weighed 2.9 to 4.0 kg (mean = 3.4 kg). Rabbits were provided a Kliba Nafag 3410 diet (Kliba Nafag, Kaiseraugst, Switzerland) ad libitum. Thirty-four animals were initially assigned according to the planned study design to the three study groups, as outlined in Table 1, and five animals were retained as spares for potential reallocation (replacement or addition) in the event of dropouts, as detailed in Supplementary S1.A.

**Table 1.** Planned Experimental Design of the Rabbit Ocular Study With Pre-Assigned Animal Identifiers; Deviations From This Plan Are Detailed in Supplementary S1.A

Group	Description	*N*	Animal ID	Number of Doses	Item	Dose Volume	Dose Level OU
1	Vehicle	4	036–039	1	Vehicle	30 µL	0 mg/eye
2	Single dose	16	251–266	1	Ranibizumab	30 µL	0.5 mg/eye
3	Multiple dose	14	351–364	3	Ranibizumab	30 µL	0.5 mg/eye

OU, oculus uterque (both eyes). See Table 2 for the study schedule of activities, including the dosing interval for group 3.

A summary of study procedures and the schedule of activities for each group are provided in Table 2. All animals underwent bilateral ocular MRI within 1 week prior to dosing. Ocular biometry (A-scan ultrasound) was performed bilaterally at baseline and at termination.

**Table 2.** Schedule of Activities in the Study

		Time		
Activity	Laterality	Group 1	Group 2	Group 3
MRI	OU	Predose		
Biometry	OU	Predose and termination		
IVT dosing		OS: 0 h	OU: 0 h	OU: 0 h, 336 h, 672 h
Ocular examinations	OU	Baseline examination and at least once per week during the study (e.g. on day of AH sampling or IVT injection)		
Optical coherence tomography (OCT)	OU	Predose, 336 h, 672 h, and 1008 h (group 3 only), termination		
In vivo sampling (AH and blood)		OS: Predose and termination	OU: Sparse sampling, see Supplementary Table A1 of Supplementary S1.A	
Terminal sampling (AH, VH, blood)	OU	Staggered termination, see Supplementary Table A1 of Supplementary S1.A		
Ocular tissues for gene expression	OU	Staggered termination, see Supplementary Table A1 of Supplementary S1.A		

Group 1: vehicle; group 2: single dose of ranibizumab; group 3: three doses of ranibizumab (see Table 1). OU, both eyes; OS, left eye. For the complete schedule of in vivo AH sampling and termination times, see Supplementary Figure A1 in Supplementary S1.A.

IVT dosing was performed on day 1 for all groups, with additional injections at 14 and 28 days for group 3. Ranibizumab was obtained from Genentech (South San Francisco, CA, USA) and administered at a dose of 0.5 mg per eye, consistent with the clinical dose used in humans.^45^ This dose is consistent with doses previously used in rabbits and has been shown to be well-tolerated following both single and repeated IVT administrations.^22^^,^^46^

AH was sampled serially during the study for PK analysis, with the complete schedule presented in Supplementary Figure A1 of Supplementary S1.A. Animals were terminated in a staggered manner across 8 time points in the single-dose group and 7 time points in the multiple-dose group, extending to 12 weeks after the first injection.

Ocular safety assessments included complete ocular examinations (slit lamp biomicroscopy, intraocular pressure [IOP] measurement, and both direct and indirect ophthalmoscopy) and were performed at baseline and at least once per week during the study (e.g. on the day of AH sampling or IVT injection). Optical coherence tomography (OCT) was performed for each eye at the baseline, at the time of IVT injections, and at termination.

Additional activities, including blood sampling for the assessment of anti-drug antibodies (ADAs) in serum, were performed as part of the present study and are reported in this paper. In contrast, pharmacodynamic endpoints, including quantification of VEGF concentrations in aqueous and vitreous humour and gene expression in ocular tissues, were collected as part of the study design but are not included in the present analysis.

Detailed descriptions of MRI acquisition and processing, OCT acquisition, IVT injections, PK sampling, and terminal assessments are provided in the following sections. The ocular biometry methodology is detailed in Supplementary S1.A; these data were not included in the present analysis based on a posteriori evaluation.

### In Vivo Procedures

#### Magnetic Resonance Imaging

MRI was performed on a 7 Tesla/40 cm Bruker Biospec horizontal-bore small-animal scanner (Bruker BioSpin, Ettlingen, Germany), equipped with a 154 mm bird-cage resonator for excitation and a 30 mm actively decoupled, single-turn surface receiver coil. Individual anaesthetized animals were transferred to the patient bed of the MRI scanner which was equipped with a heating blanket feedback-regulated via a rectal thermometer and a face mask with tooth bar for stereotaxic fixation of the head and continuous supply of oxygen enriched air (40%). A pressure sensor placed underneath the animal's chest and a capnometer sensor line incorporated in the facemask served for continuously monitoring the animal's breathing rate and oxygenation on a PowerLab data acquisition system (ADInstruments, Spechbach, Germany). The MRI detector coil, which was mounted on a flexible arm, was initially placed around the left eye and the patient bed with the animal was positioned in the scanner. Two fast gradient-echo scout images for localizing the eye and further scan planning were acquired. Subsequently, the actual volumetric scans were performed with a set of four 3D-balanced steady-state free precession (bSSFP) acquisitions with a 25.6 mm^3^ field of view, 5.0/2.5 ms repetition time (TR)/echo time (TE), a 256 × 256 × 64 data matrix resulting in a nominal resolution of 0.1 mm × 0.1 mm × 0.4 mm, and a total scan time of 4 × 40 seconds. Thereafter, the bed was retracted from the scanner, the MRI detector coil was pivoted to the right eye and the above sequence of procedures was repeated on the second eye. For each eye, the 4 image sets acquired with phase advances of 0 degrees, 90 degrees, 180 degrees, and 270 degrees, respectively, were combined using the pixel-wise root-sum-of-squares method, effectively removing the banding artifacts typically seen in bSSFP imaging.

#### MR Image Processing and Analysis

On the preprocessed image volumes, the rough outline of the eye, a seeding area in the lens, and the boundary cuts between the vitreous, iris/ciliary body, and anterior chamber were drawn manually. Subsequently, an automatic segmentation within the initial masks was performed using MATLAB's “activecontours” function (The MathWorks Inc., Natick, MA, USA), yielding 3D outlines of the vitreous, lens, anterior chamber, and the iris/ciliary body. The volumes of the segmented compartments were computed by counting the number of image voxels within the individual compartment's mask and multiplied by the volume of a unit voxel.

In preparation for automatic and accurate determination of linear dimensions, all images were centered and registered to the optical axis. In a first step, the centers of gravity (COGs) of the three largest compartments, that is, vitreous, lens, and anterior chamber, were determined using MATLAB's “regionprops3” function. The position of the optical axis of the eye was then inferred from the COGs by means of a principal component analysis using MATLAB's “pca” function. A rigid body image transformation was performed to align the optical axis with the standard orientation. Finally, linear dimensions were determined by bounding box analysis of the segmented volumes to extract and average their maximum extents along and perpendicular to the optical axis on the central two slices flanking the optical axis of the registered data set. The resulting accuracy of the linear measurements was approximately 0.05 mm. The linear dimensions of interest are schematized in Figure 1 and were the vitreous diameter (VD; see Fig. 1A), the lens diameter (see Fig. 1B), the anterior chamber depth (see Fig. 1C), the lens thickness (see Fig. 1D), the vitreous chamber depth (see Fig. 1E), and the optical AL (see Fig. 1F).

**Figure 1.**
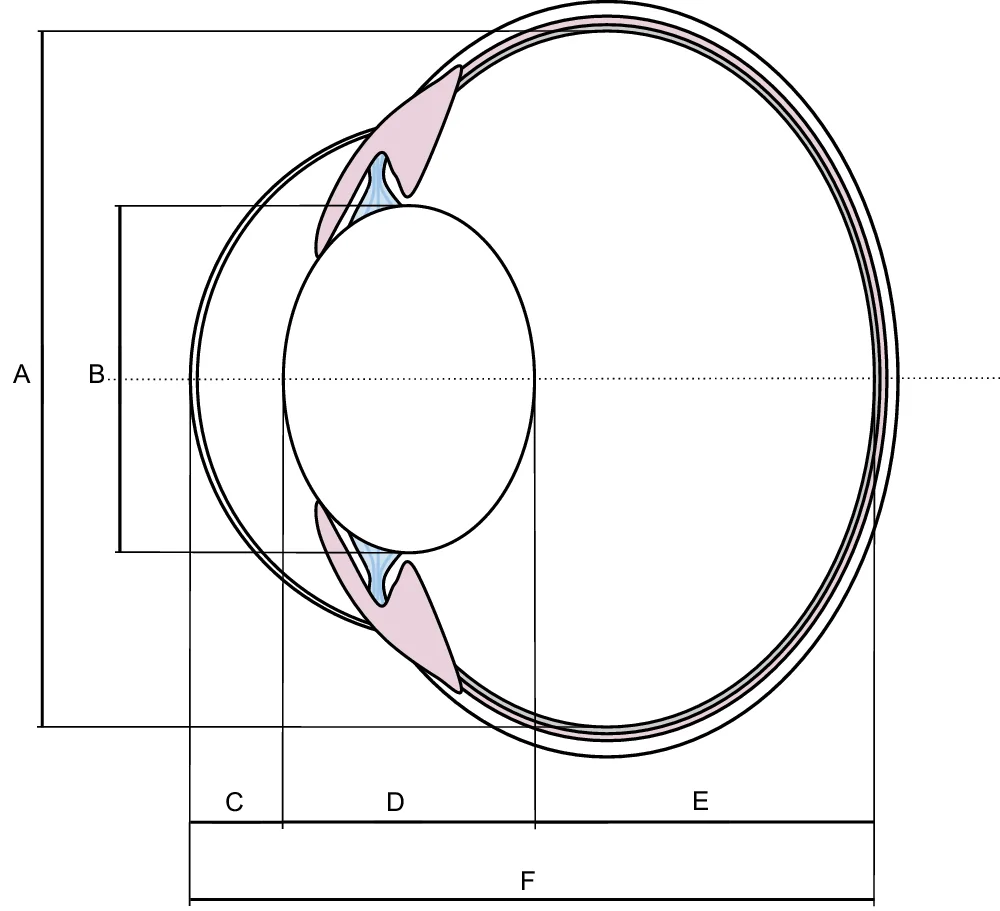
Schematic of the rabbit eye and of the key measurements estimated from the MRI study. The *dashed line* corresponds to the optical axis. See text for further details.

#### Intravitreal Injection and Sampling Procedures

Prior to IVT injection or AH sampling, anesthesia was induced with medetomidine (0.2 mg/kg, intramuscular) followed by S-ketamine (6 mg/kg) or ketamine (20 mg/kg) given subcutaneously/intramuscularly. Oxygen was provided by a face mask, and mydriasis was achieved with topical tropicamide (1% tropicamide eye drops). Atipamezole (0.5–1 mg/kg, intramuscular) was administered to reverse anesthesia after the procedure.

Following induction of anesthesia, animals in groups 2 and 3 received an initial IVT injection of ranibizumab (0.5 mg in 30 µL) in each eye at 0 hours; animals in group 3 received additional IVT injections at 336 and 672 hours. The eyes were prepared for IVT injection under aseptic conditions. The periocular area and conjunctival fornix were disinfected using povidone-iodine 2.5% ophthalmic solution and rinsed with sterile saline, and a topical anesthetic (tetracaine 1% eye drops) was applied. Eyelids were retracted with a speculum, and the globe stabilized with conjunctival forceps. Under a surgical microscope and using a prefilled syringe fitted with a 30-gauge needle, the injection was performed through the sclera approximately 4 to 5 mm posterior to the limbus at a 45-degree angle to the optic nerve head. After needle withdrawal, gentle pressure was applied at the injection site to minimize reflux, and a topical ocular lubricant was administered (Corneregel, Bausch+Lomb, Vaughan, Canada). Animals were monitored during recovery before being returned to their home cages.

Serial AH samples were collected from anesthetized animals using a 30-gauge needle, with a target volume of 40 µL per eye. Each sample was divided into 2 equal aliquots and transferred into PCR tubes placed in a 96-well matrix plate. Samples were stored at temperatures below −70°C until analysis.

Animals were euthanized in a staggered manner to permit collection of ocular tissue samples at different times after the last injection. Euthanasia was performed by intravenous administration of pentobarbital (150 mg/kg) into the marginal ear vein, followed by exsanguination after confirmation of death by auscultation. The eyes were enucleated and AH (target volume of 40–100 µL per eye), vitreous humour, and ocular tissues were collected; only AH samples were used in the numerical analysis. Initial inspection of the data suggested that differences in the sampling procedure and volume between terminal and in-life AH samples may have influenced the measured concentrations, rendering them unreliable for quantitative analysis. These samples were therefore excluded from the numerical analysis; further details are provided in Supplementary S1.A.

#### Optical Coherence Tomography and Confocal Scanning Laser Ophthalmoscopy Acquisition

OCT/confocal scanning laser ophthalmoscopy (cSLO) imaging was conducted with a Heidelberg Spectralis platform (Heidelberg Engineering, Heidelberg, Germany). On imaging days, one drop of a mydriatic solution (1% tropicamide eye drops) was applied to each eye to ensure adequate pupil dilation. For every eye, a single scan in high resolution settings was obtained (target settings: ART set to 100 frames; number of B scans averaged) using a scan angle of 20 degrees, extending throughout the fundus (nasal and temporal), with the image centered on the area centralis. Moreover, two volume scans at high-speed settings were performed (target settings: ART set to 40 frames; total volume of 7 B scans) with a scan angle 30 degrees, size 20 × 15. One volume scan was positioned below the visual streak, and the second one was positioned inferior to the first one. The minimum scan quality required for each single B scan was set at 25. Additional images were captured in regions of interest.

### Bioanalytical Methods

#### Quantification of Ranibizumab in Serum, Aqueous Humour, and Vitreous Humour

Ranibizumab was quantified using a homebrew Simoa-HDX two-step assay. VEGF 165 (catalog #293-VE; R&D Systems, Bio-Techne, Minneapolis, MN, USA) was covalently coated to Simoa Singleplex homebrew beads 488 (catalog #104006; Quanterix, Billerica, MA, USA) at 0.5 µg/mL. Detection was performed using an anti-human kappa light chain antibody (clone 1.7.10; Roche, Basel, Switzerland), which was diluted to 0.5 µg/mL. Streptavidin-β-galactosidase (SBG) was diluted to 150 pM/mL. A calibration curve of ranibizumab (Lucentis, Novartis, Basel, Switzerland; PZN-16797532) was prepared in sample diluent (catalog #101359; Quanterix) in the range from 1500 pg/mL to 2 pg/mL. Serum, AH, and vitreous humour samples were diluted at least 1 to 100 and preincubated with the beads and detection antibody. The beads were then washed and the SBG was added to the beads. After another washing step, the beads were separated and, by adding resorufin-β-galactopyranoside, light was released in the presence of SBG, which was captured by a high-resolution camera. A four-parameter fit in XLfit (IDBS, Guildford, UK) was used to calculate the sample concentrations based on the standard curve.

#### Determination of Anti-Ranibizumab Antibodies

ADAs against ranibizumab were detected in serum samples using a bridging ADA assay. Biotin- and digoxigenin-labeled ranibizumab (Roche Diagnostics, Penzberg, Germany) were preincubated at a concentration of 1 µg/mL with 1:50 diluted serum samples. In the presence of antibodies against ranibizumab, these were able to bridge the 2 differently labeled ranibizumab molecules. The immune complex was captured on a streptavidin microtiter plate (Microcoat Biotechnologie, Bernried, Germany) via the biotin and, after a wash step, it was labeled using an anti-digoxigenin horseradish peroxidase (HRP) antibody. After another wash, the HRP caused a color reaction through addition of 2,2′-azino-di(3-ethylbenzthiazoline-6-sulfonic acid) (ABTS) as a substrate. An affinity-purified monoclonal mouse antibody (clone 2.45.51; Roche, Basel, Switzerland) was used as a positive control in three quality control (QC) concentrations on each measured plate (HQC = 100 ng/mL, MQC = 10 ng/mL, and LQC = 1 ng/mL). Reagents and samples were incubated at room temperature with shaking at 500 rpm. Roche Universal Buffer was used as the assay buffer and PBS with 0.05% Tween 20 was used as the wash buffer. The measurement was monitored at 405 nm (reference wavelength 490 nm). A study cutoff point was used to define ADA positivity, with thresholds derived from each subject's pre-dose samples.

### Construction of the 3D Ocular Models

To model vitreous ranibizumab PKs in rabbits, three mathematical models with progressively increasing anatomic realism were developed. The first model varied optical AL, the second varied both AL and the VD, and the third used reconstructed ocular geometries derived from MR images. These are referred to as the ocular biometry (AL) models, the ocular biometry (AL+VD) models, and the MRI models, respectively. Ocular half-life was subsequently computed for each geometry using the MFPT framework described in the following subsection.

#### **Model 1:** Ocular Biometry (AL) Models

To quantify the influence of population-level variation in AL, model 1 used idealized 3D vitreous chamber geometries (Fig. 2) in which only AL varied (measurement F in Fig. 1). The base geometry was constructed from mean MRI-derived measurements (see Supplementary S1.B), with variation introduced solely through AL, consistent with information available from ocular biometry.

**Figure 2.**
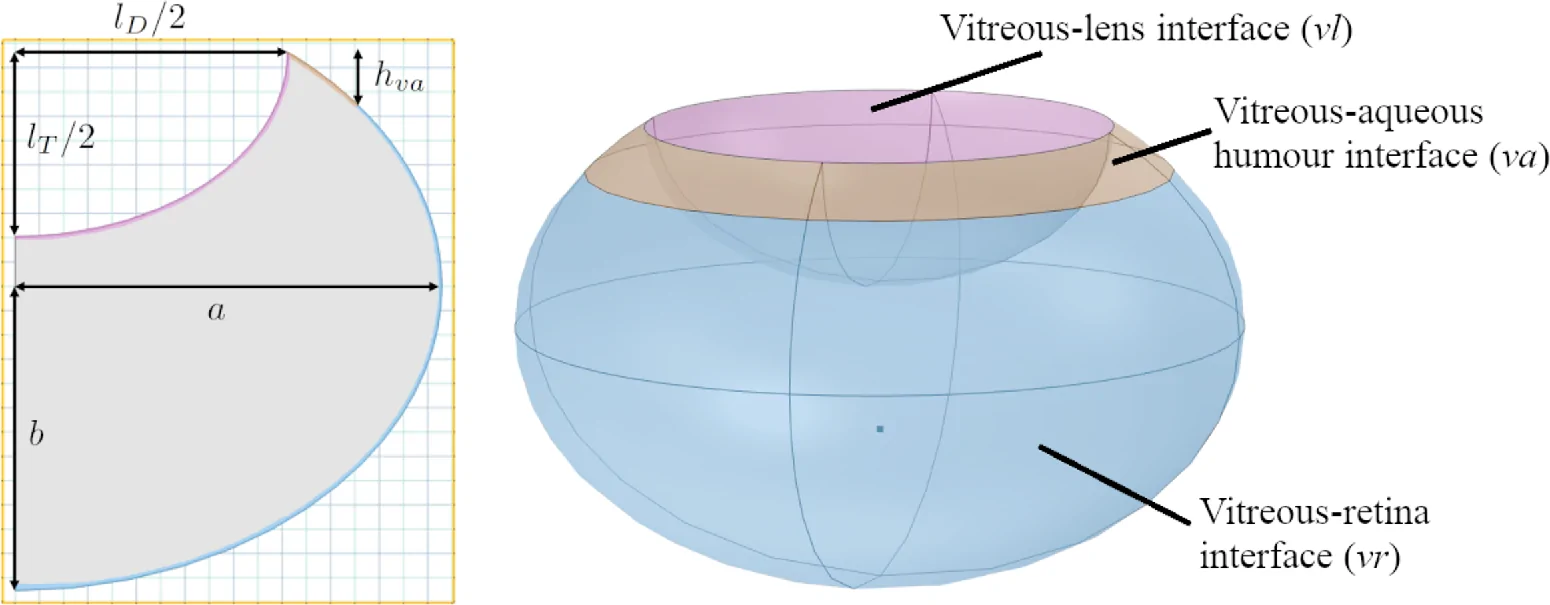
*Left*: Plane geometry used in the axial rotation to define the baseline 3D ocular biometry model, with the parameters used to construct the geometry (parameters defined in Supplementary Table B1 of Supplementary S1.B). *Right*: Vitreous body domain and its three surface interfaces: the vitreous-lens (∂Ω_*vl*_), vitreous-aqueous humour (∂Ω_*va*_), and vitreous-retina interface (∂Ω_*vr*_).

#### **Model 2:** Ocular Biometry (AL+VD) Models

As AL is generally correlated with vitreous chamber width, with longer eyes tending to have wider vitreous chambers and shorter eyes narrower ones as documented in humans,^32^ model 2 extends model 1 by allowing the VD to vary. In this model, both AL (measurement F in Fig. 1) and VD (measurement A in Fig. 1) vary concurrently according to individual biometric values, whereas the base geometry remains unchanged (see Fig. 2). This extension enables evaluation of whether AL alone is sufficient to characterize ocular geometry for accurate prediction of ocular residence time.

#### **Model 3:** MRI Models

Anatomically detailed vitreous geometries were reconstructed from the rabbit MRI dataset to generate subject-specific models of IVT drug distribution. The geometry reconstruction broadly follows established MRI-based surface-mesh generation methods^36^ and is consistent with approaches used for human ocular geometries.^37^ Details are provided in Supplementary S1.B, with the key steps illustrated in Figure 3, and validation is described in Supplementary S1.C. All generated meshes are available at the GitHub repository: https://github.com/patricia-lamirande/Individual-Eye-Pharmacokinetics-of-Ranibizumab.

**Figure 3.**
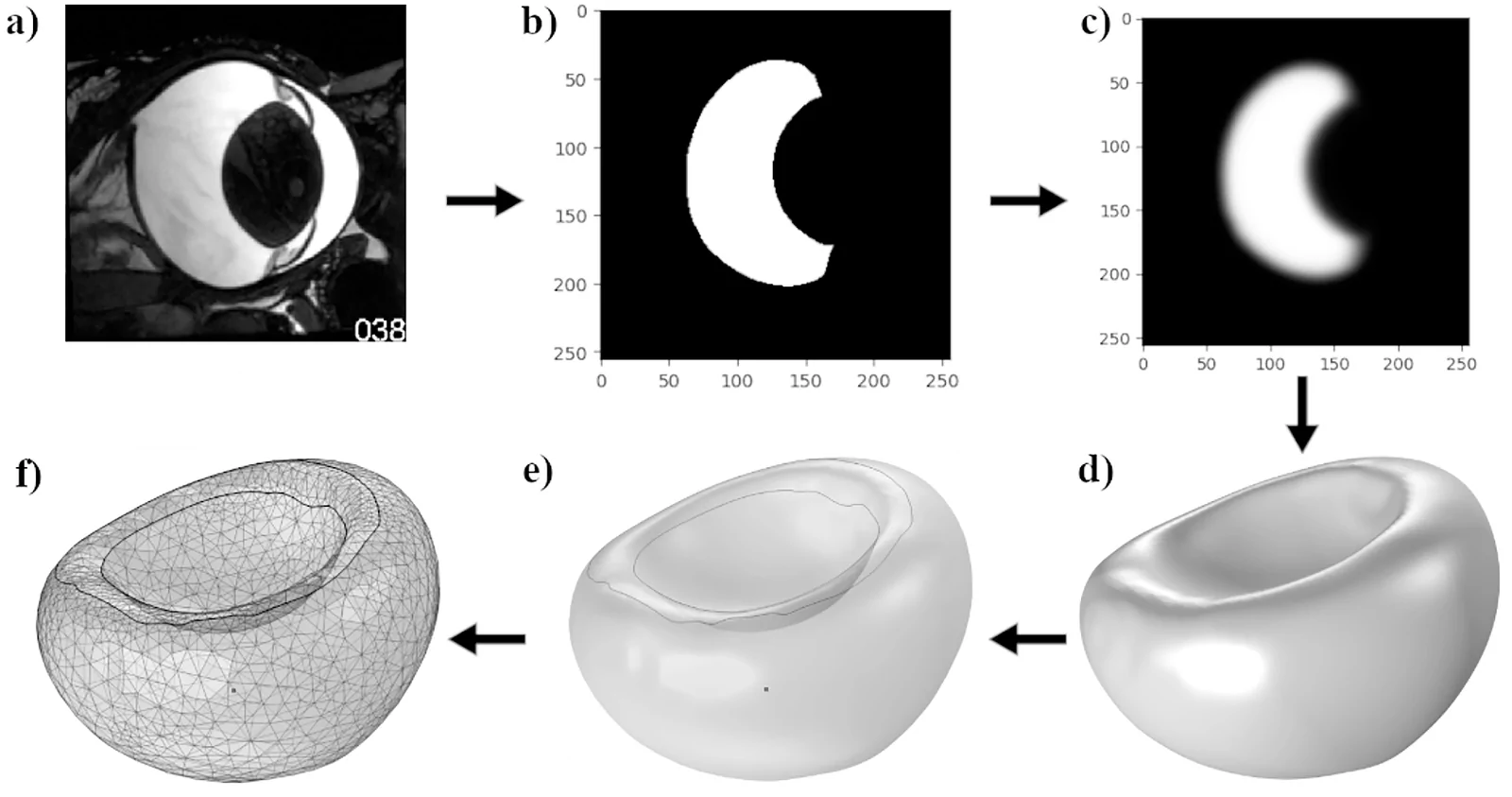
A schematic of the mesh construction methodology, using the MR image of the left eye of subject 038. (**a**) Central slice of the MR image volume, (**b**) cross-section of the constructed mask for the vitreous, (**c**) cross-section of the smoothed mask after applying a Gaussian filter, (**d**) geometry surface constructed using the marching cubes algorithm, (**e**) constructed geometry, and (**f**) evaluation point with constructed mesh for solving Equation 1. The methodology is further detailed in Supplementary S1.B.

#### Injection Points

In all three models, an injection location was defined at the midpoint between the lens and the retina along the visual axis, referred to as the midpoint of the vitreous depth (or simply the midpoint). This location served as a consistent reference point for comparing simulation results across eyes and models; its geometric definition is provided in Supplementary S1.B.

A second, more superficial injection depth within the vitreous chamber was considered in the ocular biometry (AL) models, representing typical injection depth used to minimize the risk of lens injury in animals with smaller eyes. This location, referred to as the side point, was included only in the ocular biometry (AL) models for simplicity and was used to assess the impact of injection location within the simpler model framework. Its definition and an illustration of both injection locations in the base geometry are provided in Supplementary Figure 3B of Supplementary S1.B.

### Mathematical Model for Mean First Passage Time Simulations

The MFPT has previously been used to quantify ocular drug retention in various species.^15^ The MFPT, defined as τ(***x***_**0**_) for a particle starting at ***x***_**0**_, satisfies the following partial differential equation (PDE):

(1) $$\begin{array}{c} -D\nabla^{2}\tau \left(x_{0}\right)=1,\;\mathrm{for}\;x_{0}\in \Omega , \end{array}$$

with boundary conditions

(2) $$\begin{array}{c} \begin{array}{cc} \nabla \tau \left(x_{0}\right)\cdot n=0\; & \;\mathrm{for}\;x_{0}\in \partial \Omega_{vl},\\ \tau \left(x_{0}\right)=0 & \;\mathrm{for}\;x_{0}\in \partial \Omega_{va},\\ -D\nabla \tau \left(x_{0}\right)\;\cdot n=\;\kappa_{vr}\;\tau \left(x_{0}\right) & \;\mathrm{for}\;x_{0}\;\in \partial \Omega_{vr}, \end{array} \end{array}$$

where *D* is the diffusion coefficient, κ_*vr*_ the permeability parameter for the vitreous-retina interface, and ***n*** the outward normal, for the vitreal region Ω and associated lens, anterior and retinal boundaries ∂Ω_*vl*_, ∂Ω_*va*_, and ∂Ω_*vr*_ respectively, as illustrated in Figure 2 for the ocular biometry base geometry. The vitreous-AH interface was treated as an absorbing boundary, allowing unrestricted drug elimination into the AH, whereas the vitreous-retina interface was modeled as semi-permeable boundary permitting restricted elimination through the posterior membrane.

The diffusion coefficient was set to *D* = 1.07  ×  10^−10^ m^2^/s, corresponding to the mean value reported for molecules of similar size to ranibizumab.^13^ The vitreous-retina interface permeability was defined as the lowest permeability between the inner limiting membrane and the retinal pigment epithelium, with κ_*vr*_ = 1.81  ×  10^−9^ m/s for ranibizumab, as previously estimated using mathematical modelling.^47^ These parameter values are consistent with our previous work.^15^ The governing equations were discretized using finite element methods and solved with a stationary solver in COMSOL Multiphysics (COMSOL AB, Stockholm, Sweden) for each geometry defined in the previous section.

### Pharmacokinetic Model of Ranibizumab Ocular Elimination

Based on experimental observations that ranibizumab is removed at similar rates in the aqueous and vitreous humours, the elimination rate constant was estimated from AH samples and assumed to represent overall ocular elimination.^20^^,^^22^ The drug concentration was assumed to follow an exponential decay after each dose:

(3) $$\begin{array}{ccc} C\left(t\right) & = & \frac{m}{V_{0}}e^{-\lambda \;\left(t-t_{1}\right)}\cdot 1_{t\;>\;t_{1}}+\frac{m}{V_{0}}e^{-\lambda \;\left(t-t_{2}\right)}\cdot 1_{t\;>\;t_{2}}\\ & & +\frac{m}{V_{0}}e^{-\lambda \;\left(t-t_{3}\right)}\cdot 1_{t\;>\;t_{3}}+... \end{array}$$

where *C*(*t*) is the quantity of the drug in the ocular compartment at time *t*, *t_i_* is the time of the *i*th injection ($t_{1}<t_{2}<...$), *m* is the injected mass, *V*_0_ is the apparent ocular volume used to determine the initial concentration after administration, and λ is the elimination rate constant. The indicator function is given by $1_{t\;>\;t_{i}}=1$ if *t* > *t_i_*, and zero otherwise.

Note that the parameter *V*_0_ does not correspond to the anatomic volume of AH, but rather to an effective volume arising from coupling between the vitreous and AH compartments that governs the peak concentration of the post-transient exponential decay. In this two-compartment model, *V*_0_ arises from the quasi-steady-state solution of the governing ordinary differential equations and can be expressed as *V*_0 _ =  *V_vh_* · *CL_ah_*/*CL_dif_*, where *V_vh_* is the vitreous volume, *CL_ah_* the AH clearance, and *CL_dif_* the diffusion-limited clearance across the vitreous-AH interface.^48^ Using *CL_dif_*  =  λ · *V_vh_*, this simplifies to

(4) $$\begin{array}{c} V_{0\;}=CL_{ah}/\lambda . \end{array}$$

Both *V*_0_ and λ were obtained by fitting Equation 3 to the experimental data, as detailed below.

The ocular half-life, *t*_1/2_, is defined by the post-transient elimination rate constant λ and is independent of the dosing regimen. For the single-dose group, *t*_1/2_ is obtained from Equation 3 by considering only the first dose and determining the time required for the initial concentration to decrease by half,

(5) $$\begin{array}{c} t_{1/2}=\frac{ln2}{\lambda }. \end{array}$$

The same definition applies to the multi-dose group by considering the decay phase following the final dose.

As shown in our previous modeling work,^15^ *t*_1/2_ can be related to the MFPT, τ, through the relation

(6) $$\begin{array}{c} t_{1/2}=\ln\left(2\right)\;\tau . \end{array}$$

In Equation 6, the IVT bolus is assumed to be delivered exactly at the vitreous depth midpoint, such that τ is independent of the injection location ***x***_**0**_. Consequently, injection-site-dependent variations in the MFPT are neglected, as they cannot be resolved from the PK data. Equation 6 was used to compare MFPT predictions obtained from Equation 1 with boundary conditions (2) to experimental PK measurements.

### Mathematical Methods for Parameter Estimation

The profile likelihood method was used to calculate the maximum likelihood estimate (MLE) of parameters, derive confidence intervals (CIs), and assess identifiability.^49^^–^^55^ Ranibizumab concentration data were log-transformed prior to analysis. Assuming exponential decay (Equation 3), the model was fitted on the log scale.

Let *y*(*t*) denote the measured concentration at time *t*. We assumed

$$\begin{array}{c} ln\left(y\left(t\right)\right)∼N\left(ln\left(C\left(t\right)\right),\;\sigma^{2}\right), \end{array}$$

where *C*(*t*) is the model-predicted concentration (from Equation 3) and σ^2^ a constant error variance. Normality and homoscedasticity (constant variance for the error terms) assumptions were verified in Supplementary S1.D.

The profile likelihood framework was implemented with free parameters [*V*_0_, λ], where *V*_0_ (apparent ocular volume) was estimated for each eye and λ (elimination rate constant) was the parameter of interest used to derive *t*_1/2_. The residual standard deviation was fixed at σ = 0.26, as derived from the residual analysis in Supplementary S1.D. This corresponds to a geometric standard deviation of 1.30 on the original scale, indicating that observed concentrations were typically within 30% of model predictions.

Terminal samples were excluded due to concerns regarding reliability (see Supplementary S1.A). Observations below the lower limit of quantification (LLQ) were handled using the M1 method,^56^ in which such observations are omitted. This approach is appropriate when the proportion of observations below the LLQ is low (<30%),^57^ as in the present study. Only subjects with at least three valid observations (excluding LLQ and terminal samples) were included in the analysis.

Exploratory nonlinear mixed-effects modeling was also performed using Monolix^58^ to assess the structure of inter- and intra-animal variability and to support population-level inference (see Supplementary S1.G for details).

## Results

### Imaging Results

MRI-derived ocular biometric measurements for all subjects are shown in Figure 4, with full individual results reported in Supplementary S3. Lens thickness and anterior chamber depth exhibited limited variability, whereas the VD, optical AL, and vitreous volume showed a broader distribution. The distribution of AL and vitreous volume are further analyzed in Supplementary S1.E; for both, the hypothesis of normality was not rejected at a significance level of 0.05. Outliers in these parameters were identified and, based on imaging assessment, attributed to biological rather than technical factors. Consequently, both eyes of subject 152 were excluded from population-level analyses, as detailed in Supplementary S1.E.

**Figure 4.**
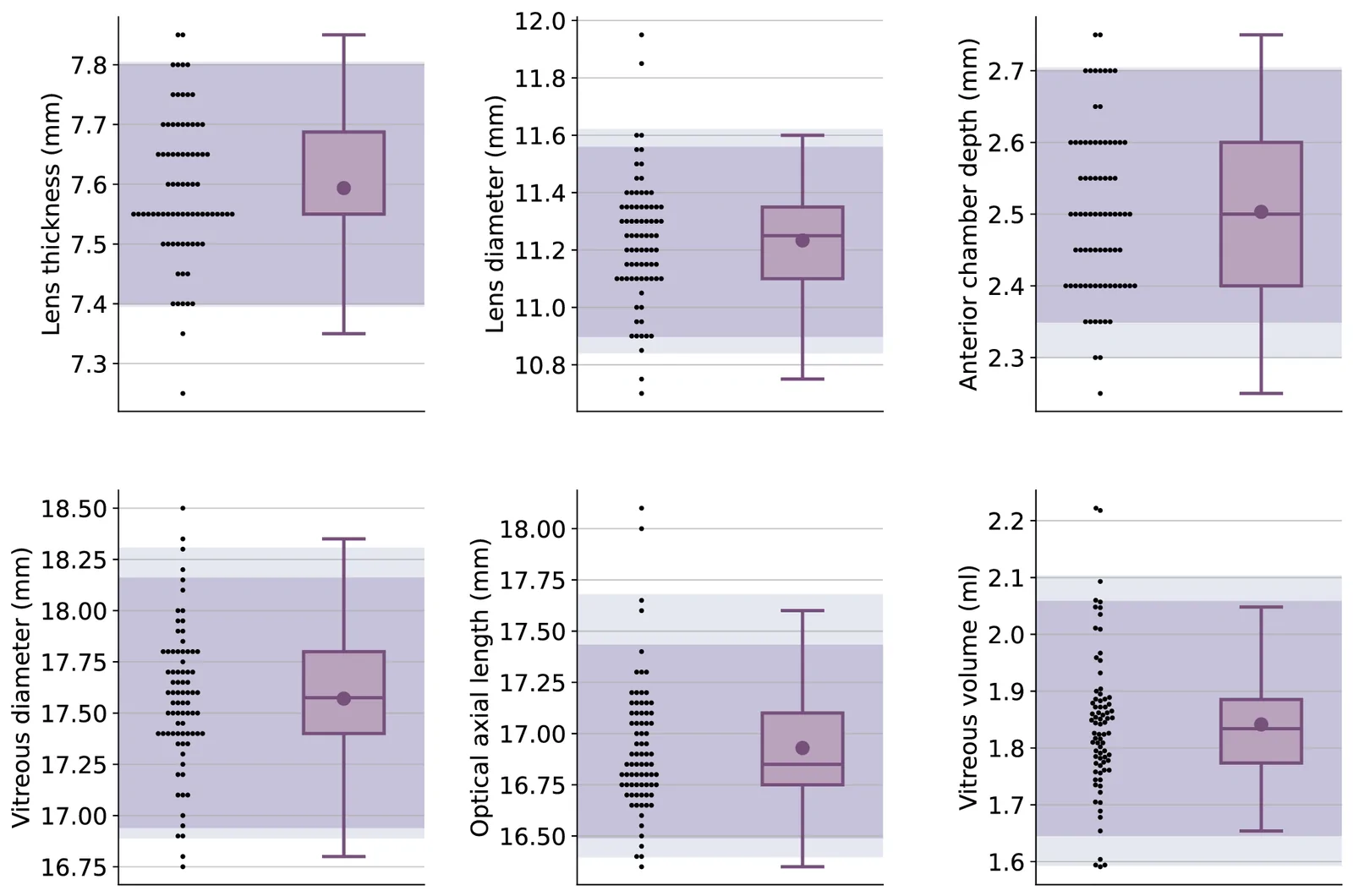
Distribution of MRI-derived ocular measurements. Raw measurements (*black points*) are shown alongside the quartiles (box and median line) and the mean (*purple point*). The central 90% (5th–95th percentiles, *light purple*) and 95% (2.5th–97.5th percentiles, *light gray*) intervals are indicated. Vitreous volume was estimated directly from MRI data, whereas linear measurements were obtained after image registration with a precision of 0.05 mm, as detailed in the text.

### MFPT Model Predictions

The MFPT was computed for the ocular biometry (AL), ocular biometry (AL+VD), and MRI models for each eye, and converted to ocular half-life, (*t*_1/2_), using Equation 6. An example is shown in Figure 5 for the left eye of subject 038, where *t*_1/2_ is plotted as a function of injection location for the ocular biometry (AL) and the MRI models. The ocular biometry (AL+VD) is not shown, as it yielded no discernible differences from the AL model. The spatial distributions of *t*_1/2_ were similar across models, with comparable ranges and contour patterns. The main difference was a slightly lower maximum half-life in the MRI model (3.2 days) compared with the ocular biometry models (3.6 days). In all models, posterior injection locations were associated with longer half-lives, whereas anterior injection locations yielded shorter values. Cross-sectional profiles (see Fig. 5c) for the example eye confirm the close agreement between the ocular biometry (AL) and MRI model, whereas additional slices of the MRI model solution (see Fig. 5d) highlight its non-axisymmetric geometry.

**Figure 5.**
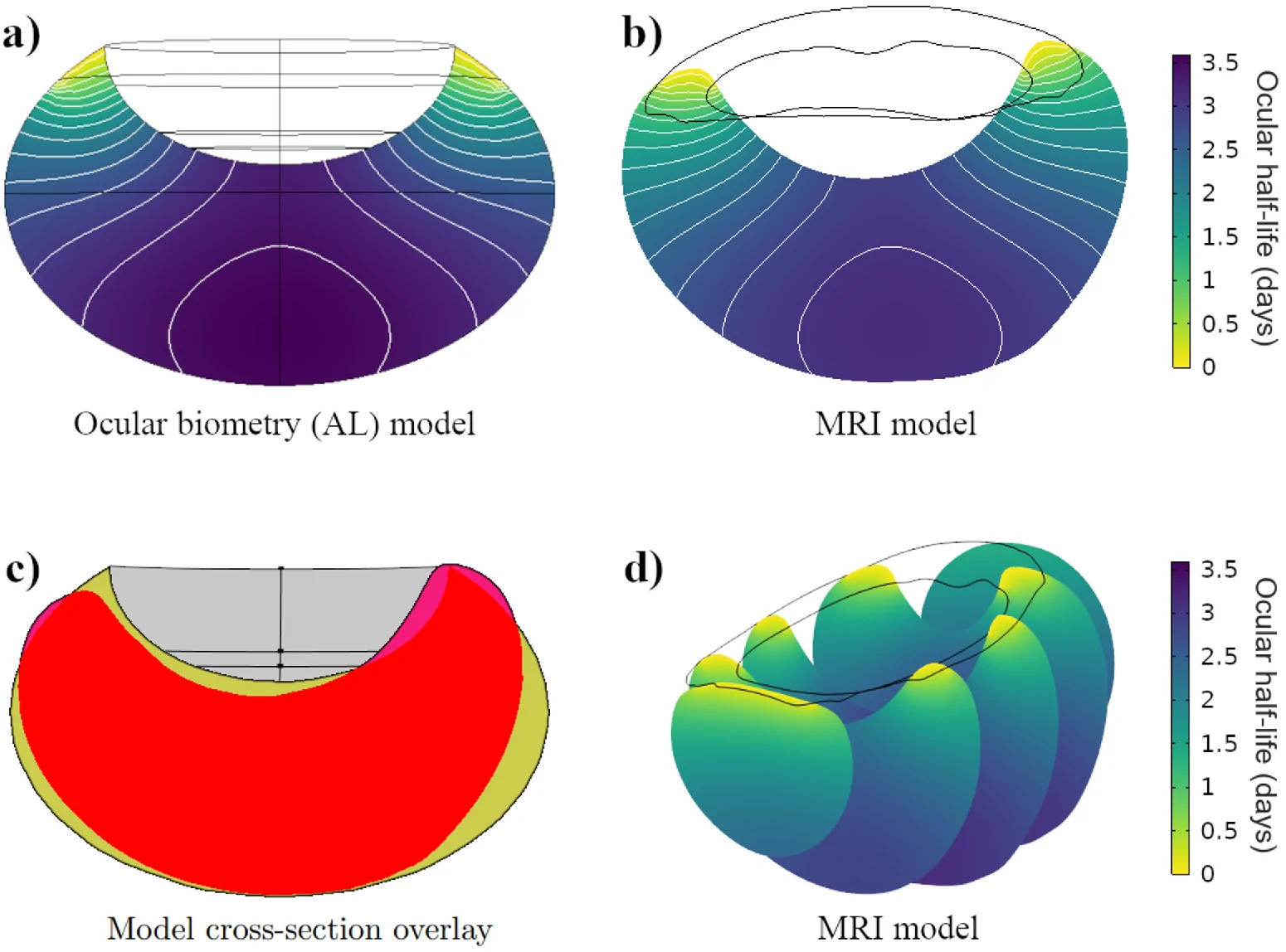
Central slice of the MFPT solution (Equation 1 with boundary conditions Equation 2) as a function of injection location for the left eye of subject 038, converted into ocular half-life using Equation 6. Results are shown for (**a**) the ocular biometry (AL) model and (**b**) the MRI model. Contours are shown in *white*, and the *black lines* are associated with the construction of the geometries and their boundaries. (**c**) Cross-sectional profiles aligned at the vitreous depth midpoint for the ocular biometry (AL) model (*pink*) and MRI model (*yellow*), with overlapping regions in *red*. (**d**) Additional slices of the MRI model solution illustrate its non-axisymmetric geometry.

Simulation results were further analyzed across the rabbit population by comparing *t*_1/2_ at selected injection sites. In the ocular biometry (AL) models, *t*_1/2_ was evaluated at the vitreous depth midpoint and side point, introduced to evaluate the impact of injection location on the simulation results, and plotted as a function of AL (Fig. 6). Across the observed axial length range, *t*_1/2_ at the midpoint varied from approximately 3.3 to 4.2 days. Results at the side point were similar, with nearly parallel linear regressions and an approximately 0.6-day offset relative to the midpoint for each eye. The half-life was also computed for the ocular biometry (AL+VD) models, where *t*_1/2_ at the vitreous depth midpoint was compared between the two vitreous models (see Fig. 6). For a given AL, the half-life from this model also varied as a function of VD, yielding results not exactly following the linear regression. The ocular biometry (AL+VD) model exhibited a steeper dependence on eye size, with smaller eyes associated with shorter *t*_1/2_, and larger eyes with longer *t*_1/2_. The half-life values and corresponding regressions were, however, not markedly different, with considerable overlap for ALs between 16.6 and 17.2 mm, encompassing most eyes.

**Figure 6.**
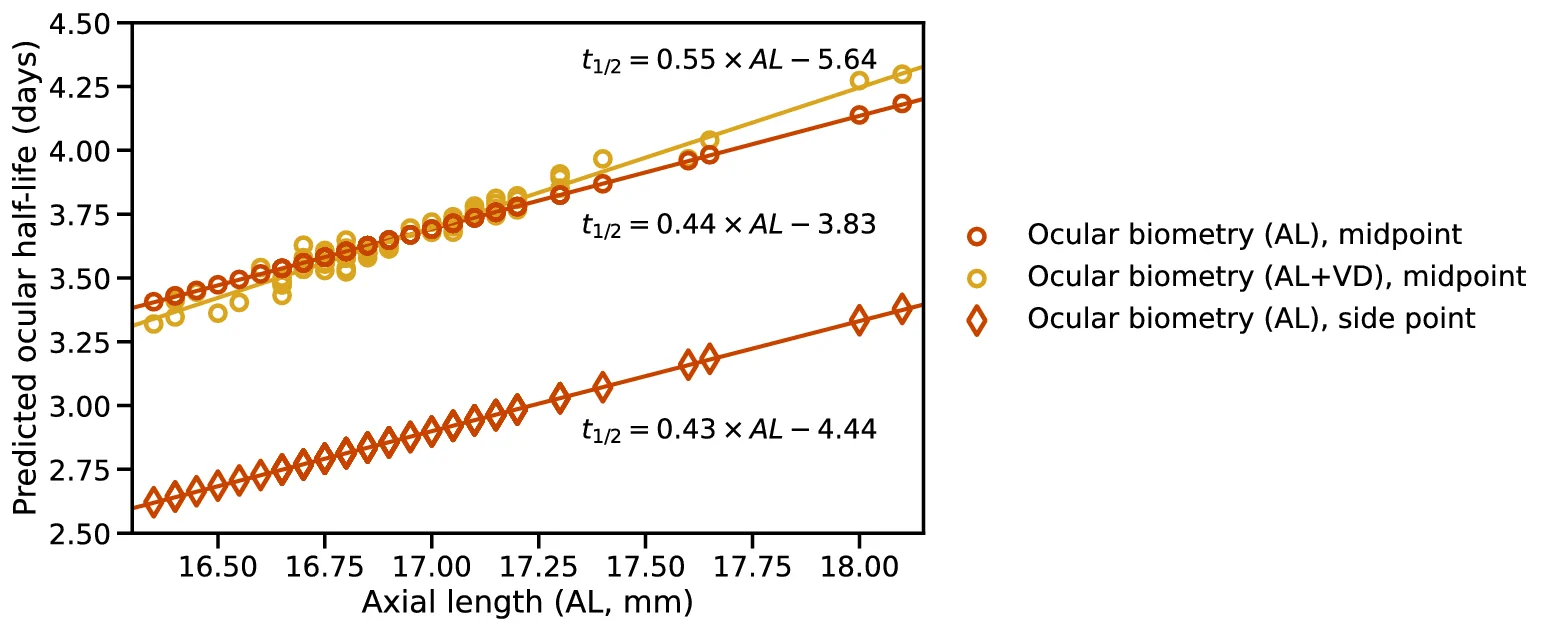
Simulation results of Equation 1 with boundary conditions (Equation 2), converted to *t*_1/2_ using Equation 6. Results are shown for the ocular biometry (AL) model at two injection locations and for the ocular biometry (AL+VD) model at the vitreous depth midpoint. Linear regression fits are also shown.

Simulation results at the midpoint were evaluated across the MRI models and plotted as a function of AL and vitreous volume (Fig. 7), together with linear regressions and coefficient of determination (*R*^2^). The simulated *t*_1/2_ ranged from approximately 3 to 4 days across the rabbit population. A strong correlation was observed between *t*_1/2_ and the vitreous volume (*R*^2^ = 0.84), whereas the correlation with AL was weaker (*R*^2^ = 0.58) and showed greater scatter around the regression line.

**Figure 7.**
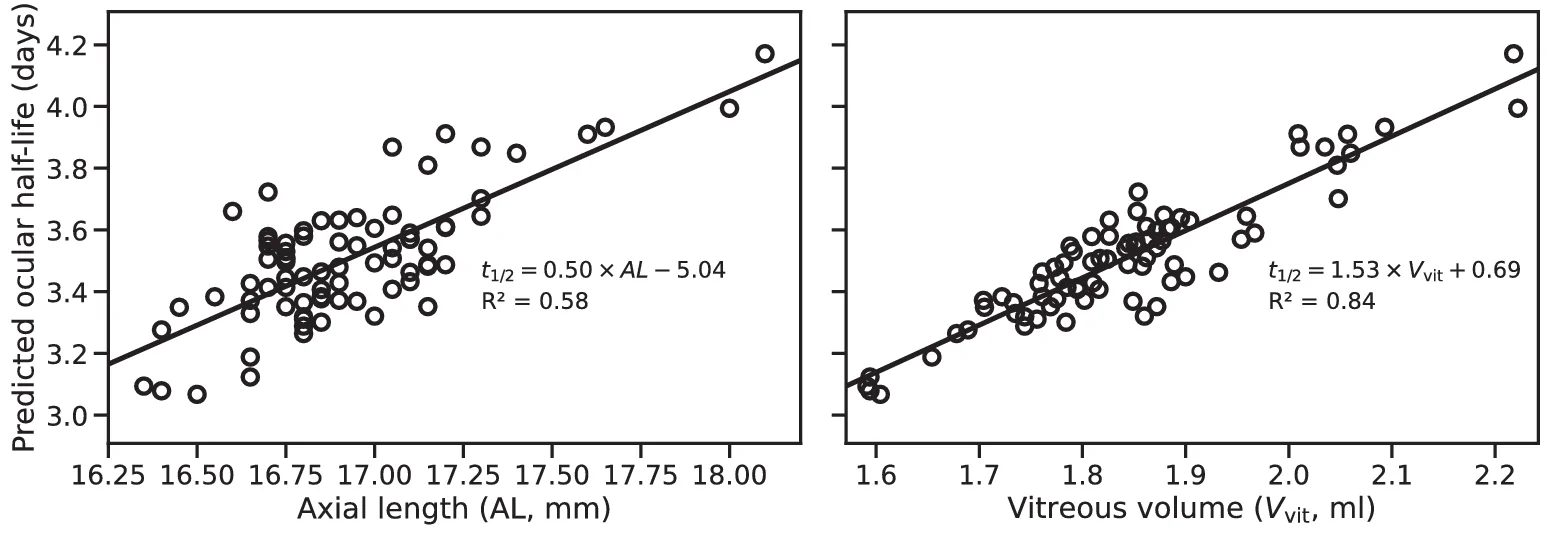
Simulation results of Equation 1 with boundary conditions (Equation 2), converted to *t*_1/2_ using Equation 6. Results are shown at the vitreous depth midpoint as a function of AL and vitreous volume for the MRI models. Linear regression fits and corresponding *R*^2^ values are shown.

Finally, the simulation results between the different vitreous models are directly compared in Figure 8, where the *t*_1/2_ (for an injection at the midpoint) in the MRI models is shown as a function of AL, along with its linear regression and the linear regressions of the ocular biometry (AL) and ocular biometry (AL+VD) models. The ocular biometry (AL) and ocular biometry (AL+VD) models tend to overestimate *t*_1/2_ compared with the MRI models, where the linear regression is approximately 0.2 days lower. The slope of 0.50 day/mm for the linear regression for the MRI models lies in between the slopes of the ocular biometry (AL) and ocular biometry (AL+VD) models, with slopes of, respectively, 0.44 and 0.55 day/mm, showing an overall good agreement between the linear regressions.

**Figure 8.**
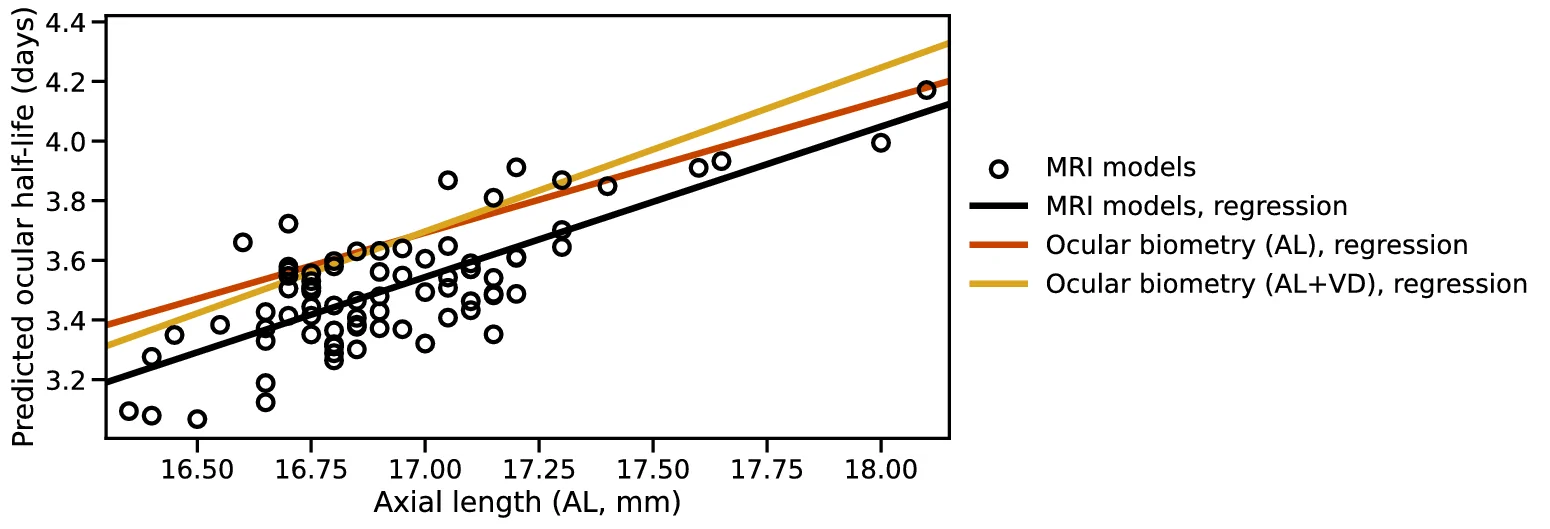
Simulation results of Equation 1 with boundary conditions (Equation 2), converted to *t*_1/2_ using Equation 6, for injections at the vitreous depth midpoint. Results for the MRI models (*black dots*) are shown as a function of AL, together with linear regression fits for the MRI (*black*), ocular biometry (AL) (*orange*) and ocular biometry (AL+VD) (*yellow*) models.

### Experimental Pharmacokinetics of Ranibizumab

Of the 34 rabbits initially assigned to the study groups (Methods), PK data were obtained from 33 animals. Two animals died or were euthanized during the study, and one was replaced using available spares. Additional reallocations between spares and study groups were performed to maintain the study design; the final group composition and animal IDs are provided in Supplementary S1.A. PK sampling yielded 378 AH samples across all time points, of which 90 (24%) were below the LLQ and were handled as described in the Methods. Terminal samples were excluded due to concerns regarding their reliability (see Supplementary S1.A). The complete dataset is provided in Supplementary S2.

Results for each eye are presented in Supplementary S1.F. Parameters *V*_0_ and λ were practically identifiable for all eyes, with MLEs and 95% CIs shown in Supplementary Figure S1F. The width of the CIs varied across eyes and was primarily driven by the number and temporal distribution of observations. Narrower CIs for λ were observed in subjects with numerous samples over a longer time after injection. For example, subjects 261 to 263 and 265 to 266 (single dose group) had 6 to 8 samples over at least 56 days after the injection, with corresponding *t*_1/2_ CIs of at most 0.8 days wide. In contrast, subjects 359, 361, and 362 (multi-dose group) had 5 samples over 28 days after the final injection, with *t*_1/2_ CIs ranging from 1.8 to 6.6 days wide. Fits of Equation 3 using MLE parameter estimates are compared with experimental observations in Supplementary S1.F, for both dosing groups, with representative examples shown in Figure 9.

**Figure 9.**
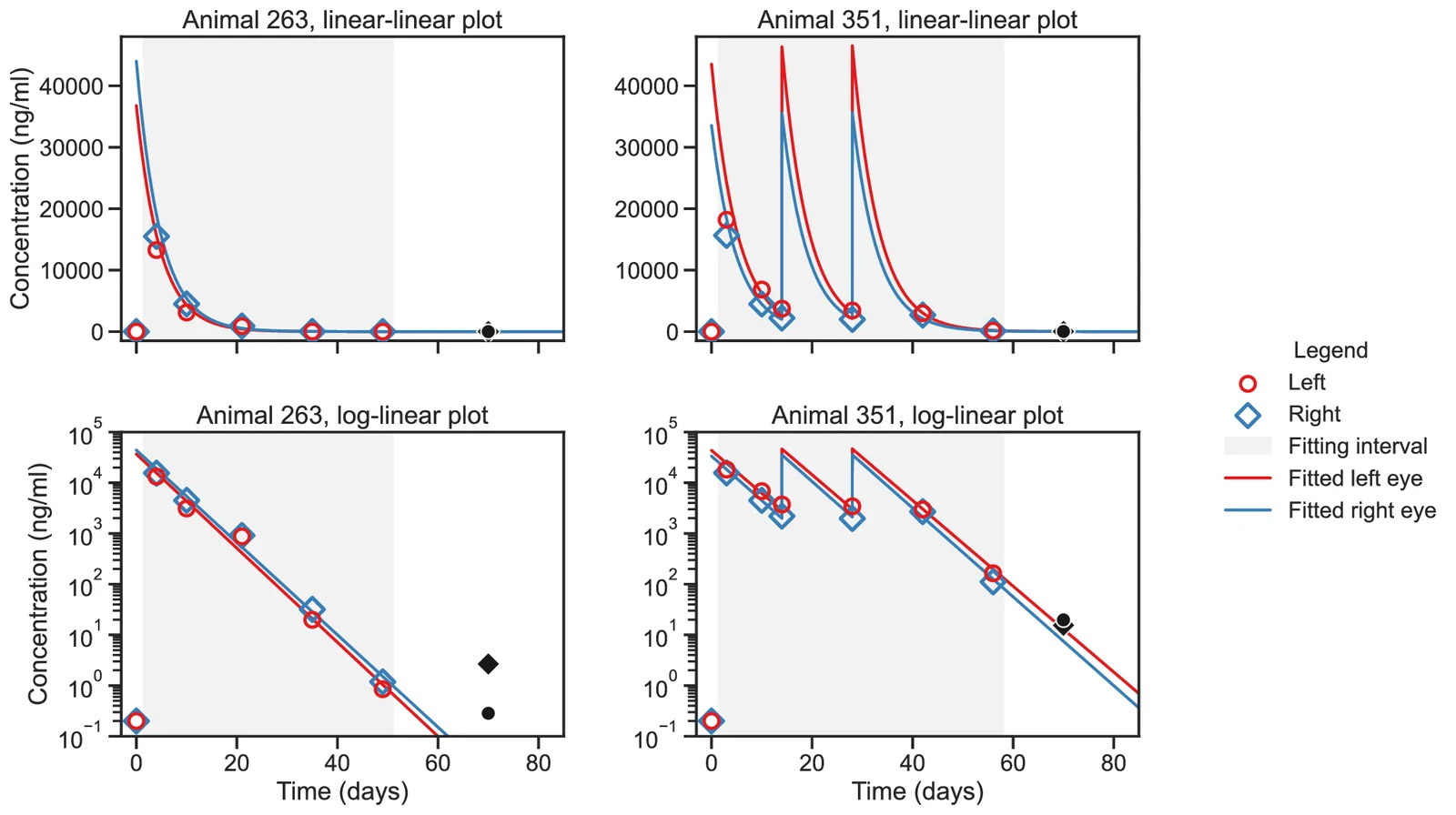
Illustrative concentration-time profiles of ranibizumab in AH for the left (*dots*) and right (*crosses*) eyes of animals 263 and 351 from the single- and multiple-dose groups, respectively. Model fits (Equation 3, MLE parameters) are shown. The *grey* region indicates data included in fitting; day 0 (pre-dose), measurements below the LLQ (plotted here at the LLQ) and terminal samples (*black* markers) were excluded. The parameter estimates and individual fits for all animals are provided in Supplementary S1.F.

The population means of *V*_0_ and λ, computed as the means of the individual MLE estimates across all eyes, were 17.1 mL and 0.189 day^−1^ respectively. Using Equation 4, this corresponds to an AH clearance of *CL_ah_*  =  2.24 µL/min, consistent with reported values of approximately 2.5 µL/min in rabbits.^59^

Ocular half-life (*t*_1/2_) was derived from λ for each eye using Equation 5, with values ranging from 2.8 to 4.9 days. Results are shown as a function of AL and vitreous volume in Figure 10a. No clear correlation was observed, with coefficients of determination near zero.

**Figure 10.**
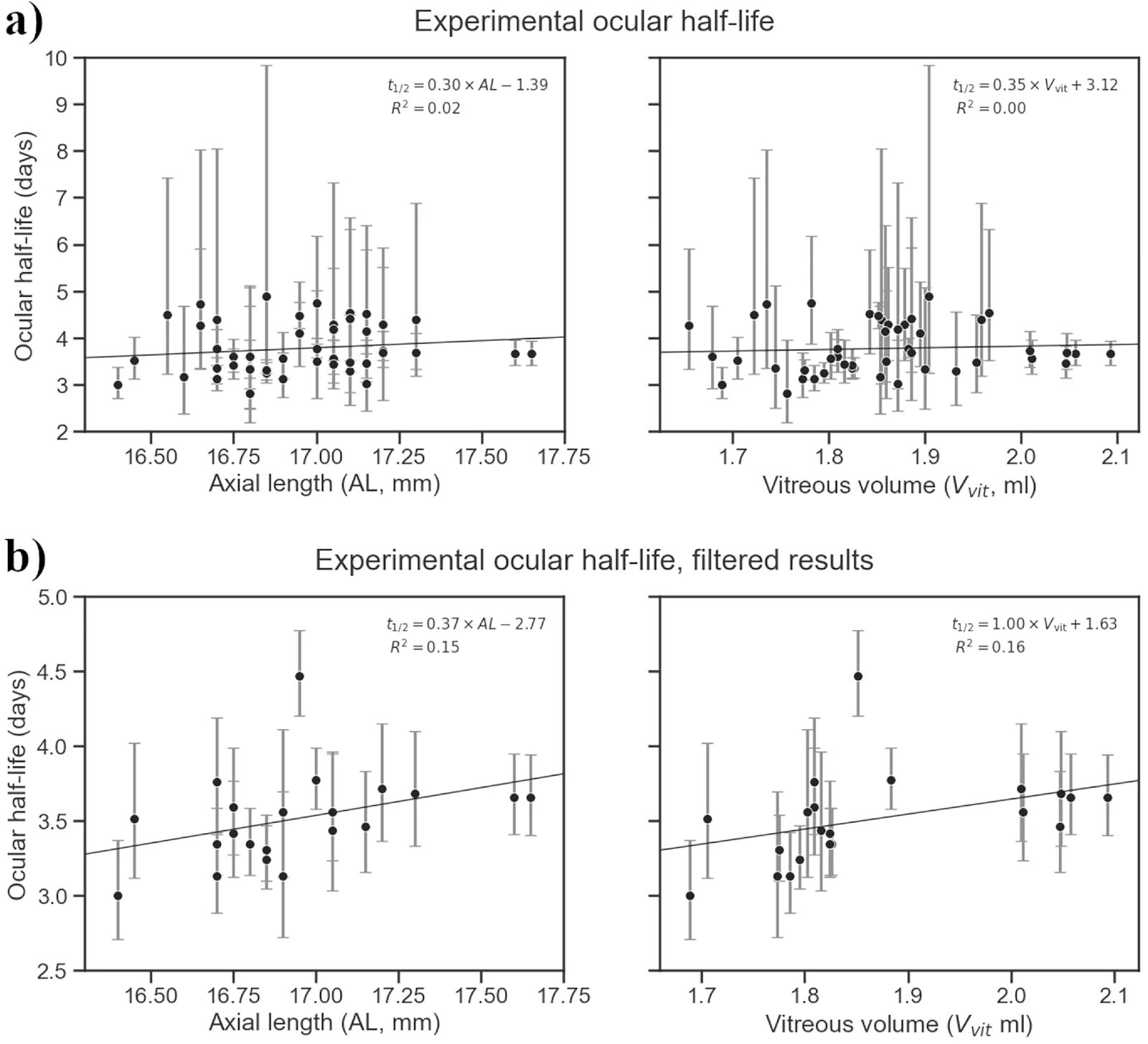
Maximum likelihood estimates (*points*) and 95% CIs (*bars*) of ocular half-life (*t*_1/2_) as a function of AL and vitreous volume. (**a**) All eyes; (**b**) estimates with CIs of at most 1 day. Linear regression fits (*black lines*) and corresponding *R*^2^ values are shown.

Because several estimates had wide CIs, a secondary analysis was performed using only *t*_1/2_ estimates with 95% CIs narrower than 1 day. This reduced the dataset to 21 eyes from 11 rabbits, corresponding to just under 50% of the total number of eyes. The retained eyes included both eyes of subjects 151, 255, 261, 262, 263, 265, 266, 351, 352, and 364, and the right eye of subject 259. Including one additional eye would require increasing the CI threshold to 1.5 days, indicating a sharp transition in estimation precision. Results for the filtered dataset are shown in Figure 10b. In this subset, a weak association between *t*_1/2_ and eye size emerged, with reduced scatter around the regression lines compared with Figure 10a. The estimated slopes were 0.37 day/mm (95% CI = 0.16–0.58) and 1.00 day/mm^3^ (95% CI = 0.47–1.53) for the AL and vitreous volume regressions, respectively.

The population mean ocular half-life was 3.67 days, derived from the mean MLE estimate of the elimination rate constant λ across all eyes using Equation 5. The corresponding standard deviation was 0.54 days, and the 5th to 95th percentile range was 3.01 to 4.66 days. These results are compared with published values in the Discussion. Results from the exploratory nonlinear mixed-effects modeling performed in Monolix^58^ are reported in Supplementary S1.G.

### Comparison of MFPT Predictions With Experimental PK Data

The MFPT predictions from the 3D MRI vitreous models are compared with experimental *t*_1/2_ values as a function of AL and vitreous volume in Supplementary S1.F. The filtered PK data, restricted to estimates with 95% CIs narrower than 1 day, is compared with simulation results from the 3 vitreous models in Figure 11. Linear regressions of ocular half-life as a function of AL for the ocular biometry (AL), ocular biometry (AL+VD), and MRI models are shown alongside the MLEs and CIs from the PK data, as well as the regression derived from the filtered PK dataset. Overall, good agreement is observed, with the regression from the filtered PK data lying among those of the three models.

**Figure 11.**
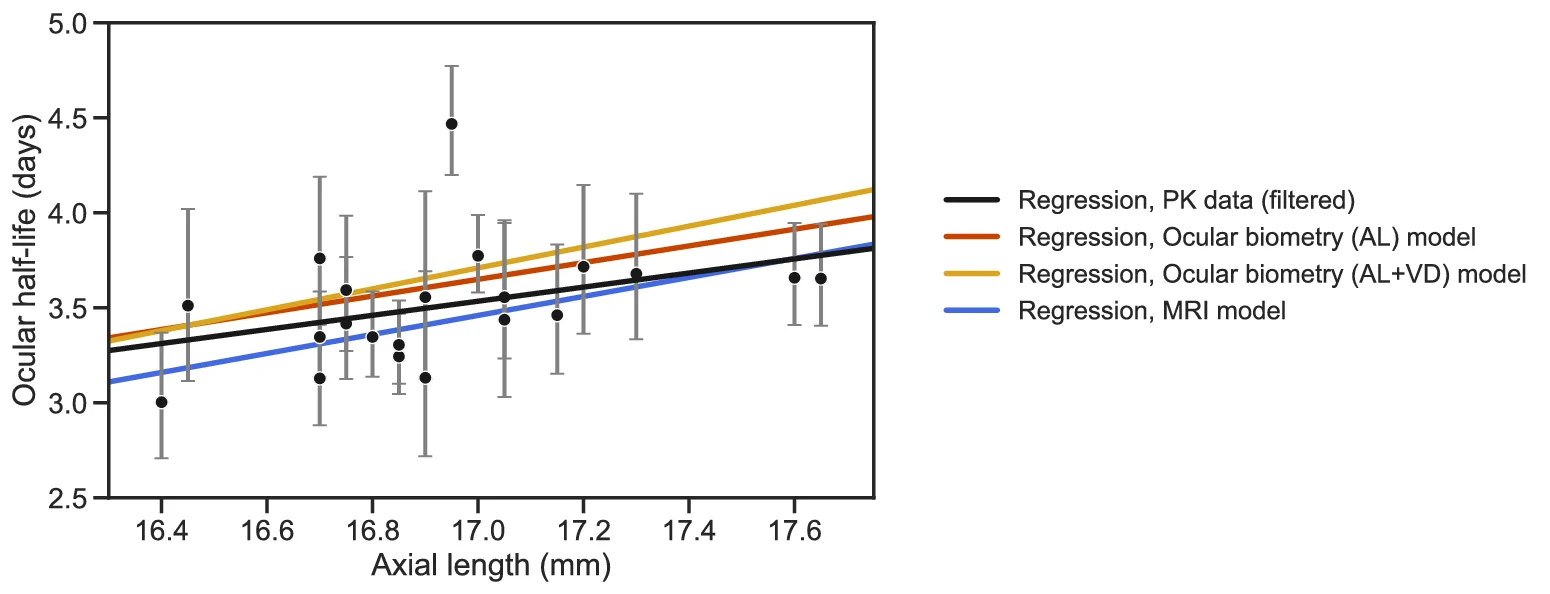
Maximum likelihood estimates (*points*) and 95% CIs (*bars*) of ocular half-life (*t*_1/2_) as a function of AL for estimates with CIs of at most 1 day. Linear regression fits for the ocular biometry (AL), ocular biometry (AL+VD), and MRI models are shown, and the regression derived from the filtered PK data. See Figures 7, 8, and 11b for the corresponding regression equations.

All approaches displayed a positive association between AL and ocular half-life. To assess whether this relationship differed by method, an analysis of covariance (ANCOVA) was performed with a linear model that allowed testing the homogeneity of regression slopes, under assumptions of linearity and homoscedasticity. No significant differences in slopes were detected across methods (*P* = 0.12). Pairwise comparisons showed that the slope of the PK data, although numerically lower, did not differ significantly from those of the modelling approaches (all *P* > 0.05).

### Serum Anti-Drug Antibody Analysis

ADAs were detected in serum in multiple animals across sampling time points (summarized in Table 3; individual animal data are provided in Supplementary S2), indicating an immunogenic response to ranibizumab under the present dosing regimen. To assess the impact of ADA formation on PKs, population-level parameters were re-estimated after excluding animals with at least one ADA-positive sample. The resulting ocular half-life (3.62 days) was similar to that obtained from the full dataset including ADA-positive animals (3.67 days), suggesting that anti-ranibizumab antibodies did not meaningfully affect drug disposition.

**Table 3.** Number of Animals With Positive Serum Anti-Drug Antibodies Against Ranibizumab per Total Number of analyzed Animals Per Time Point

	Time																	
Number of Animals With Positive ADA	0 h	48 h	72 h	96 h	168 h	240 h	336 h	504 h	672 h	720 h	768 h	840 h	1008 h	1176 h	1344 h	1512 h	1680 h	2016 h
Group 1	0/4	–	0/4	–	0/4	–	–	–	–	–	–	–	–	–	–	–	–	–
Group 2	0/17	0/2	0/4	0/6	0/9	0/6	3/8	3/6	5/6	–	–	2/4	2/4	1/3	1/2	0/2	1/2	0/2
Group 3	0/14	0/2	–	0/12	0/2	1/12	5/14	–	9/14	1/2	4/6	3/4	3/7	0/1	1/5	–	2/4	–

Group 1: (control) received vehicle (0 h), group 2: a single dose (0 h), and group 3: three doses (0, 336, and 672 h).

### Safety and Tolerability

Ocular findings related to study procedures, including AH sampling and IVT injections, were assessed by ophthalmic examinations (slit lamp, IOP, and indirect ophthalmoscopy) and OCT.

Animals in groups 2 and 3 showed findings consistent with ocular surface and intraocular inflammation following the procedures. In group 2, findings were mainly corneal neovascularization and mild to moderate anterior uveitis. A minority of eyes developed corneal erosions, vitritis, vitreous hemorrhages, or funduscopic alterations (hyporeflective lesions and scleral thinning). Based on qualitative evaluation of OCT and cSLO images, 3 of the 16 animals showed signs of minor and transient vitritis, likely related to the IVT injection. In one of these animals, potential peripheral injection site-related changes were also noted. In group 3, similar findings were observed, with increased severity after days 15 to 32 following repeated IVT injections and AH sampling. Some eyes developed severe anterior uveitis, vitritis, vitreal hemorrhages, funduscopic changes (hyporeflective lesions and scleral thinning), and reduced or lost vision. One animal was euthanized due to severe ocular inflammation. Based on qualitative evaluation of OCT and cSLO images, most animals in this group (12 of 14 rabbits) showed changes. Six rabbits exhibited signs of minor vitritis or mild vitreal hemorrhage, and four others showed signs of more severe vitreal hemorrhage. Three rabbits developed severe bilateral vitritis/chorioretinitis and vitreal hemorrhage, possibly related to the multiple injections. Additional findings included injection site-related changes. In both group 2 and group 3, the few eyes that developed corneal erosions healed uneventfully with appropriate medical treatment.

In the control group (group 1), eyes showed mild ocular surface inflammation in both untreated (OD) and manipulated (vehicle IVT injection and 2 AH samples in OS) eyes, consisting primarily of mild corneal neovascularization and mucoid discharge. None of the four animals developed any abnormalities on fundus cSLO/OCT images in either the treated or untreated eye.

IOP measurements were generally within the normal range (10–17 millimeter of mercury [mm Hg]). After AH sampling or IVT injection, a few animals displayed mild hypotony (8–9 mm Hg), whereas others showed moderate to severe hypotony (4–7 mm Hg), likely associated with procedure-induced uveitis. No episodes of elevated IOP (glaucoma) were observed.

## Discussion

This study extends an MFPT-based modeling framework to rabbits by integrating MRI-derived ocular geometries with individual-eye PK measurements, with the aim of evaluating whether inter-eye variability in ocular dimensions is associated with differences in IVT ranibizumab half-life.

Although variability in optical AL and vitreous volume has been reported for human eyes,^32^^,^^60^^–^^62^ this study represents, to our knowledge, one of the first comprehensive MRI-based quantifications of ocular geometric variability in a cohort of adult NZW rabbits (see Fig. 4). Ocular dimensions were measured in a relatively homogeneous cohort of adult NZW rabbits, thereby minimizing variability due to developmental factors. This design choice reduces confounding but also constrains the range of ocular dimensions observed. In line with previous reports,^24^^,^^25^ ocular size increases with age in rabbits, and the slightly larger values observed here (Table 4) are consistent with the older age of the cohort (23–27 weeks versus typically younger cohorts, e.g. 14–22 weeks^63^). For example, the mean AL in this study was 16.93 mm, compared with 16.31 to 16.6 mm reported in similarly sized NZW rabbits measured in vivo.^64^^,^^65^ Similarly, the mean vitreous volume of 1.84 mL exceeds values typically reported in the literature (approximately 1.06–1.7 mL; see Table 4), consistent with the use of older animals. In NZW rabbits, ocular growth has largely stabilized by 23 to 27 weeks of age,^24^ and no substantial changes in eye size were therefore expected over the course of the study.

**Table 4.** Comparison of Ocular Measurements From the Present MRI Study With Values Reported in the Literature

	Study Measurement		Comparison With the Literature			
	Mean (SD)	Range	Mean (SD)	Range (If Reported)	Reference	Details
Lens diameter, mm	11.23 (0.22)	10.70–11.95	9.95 (0.24)		[65]	*n* = 10 (NZW, 2.8–3.2 kg, sex NR), in situ
			10.30 (0.48)	9.19–11.43	[69]	*n* = 100 (mixed species, avg 2.4 kg, avg 41 mo, 21 F, 29 M), in situ, CT
			10.47 (0.31)		[65]	*n* = 10 (NZW, 2.8–3.2 kg, sex NR), ex situ
Lens thickness, mm	7.59 (0.12)	7.25–7.85	6.36 (0.13)		[65]	*n* = 10 (NZW, 2.8–3.2 kg, sex NR), ex situ
			6.9	6.8–7.0	[63]	*n* = 5 (NZW, 14–22 wk old, M), ex situ
			6.97 (0.07)		[64]	*n* = 12 (NZW, 3.2–4.2 kg, sex NR), in situ, ultrasonic
			7.85 (0.45)	6.21–9.21	[69]	*n* = 100 (mixed species, avg 2.4 kg, avg 41 mo, 21 F, 29 M), in situ, CT
			7.9	7.0–8.6	[67]	*n* = 12, (unknown), ex situ
Anterior chamber depth, mm	2.50 (0.12)	2.25–2.75	2.161 (0.110)		[65]	*n* = 10 (NZW, 2.8–3.2 kg, sex NR), ex situ
			2.34 (0.04)		[64]	*n* = 12 (NZW, 3.2–4.2 kg, sex NR), in situ, ultrasonic
Vitreous diameter, mm	17.57 (0.34)	16.75–18.50	18.0 (0.4)		[34]	*n* = 8 (NZW, 1.5–2.7 kg, M), in situ MRI
			18.03 (0.81)	16.02–19.76	[69]	*n* = 100 (mixed species, avg 2.4 kg, avg 41 mo, 21 F, 29 M), in situ, CT
			18.65 (0.29)		[65]	*n* = 10 (NZW, 2.8–3.2 kg, sex NR), ex situ
Axial length, mm	16.93 (0.31)	16.35–18.10	14.96 (0.46)	14.00–15.66	[66]	*n* = 22 (NZW, 2–3 kg, 5 moold, F), ex situ
			15.33 (0.50)	14.57–16.30	[66]	n = 18 (NZW, 2–3 kg, 5 mo old, M), ex situ
			16.0 (0.4)		[34]	*n* = 8 (NZW, 1.5–2.7 kg, M), in situ MRI
			16.31 (0.13)		[64]	*n* = 12 (NZW, 3.2–4.2 kg, sex NR), in situ, ultrasonic
			16.6		[24]	*n* = 14 (NZW, 23–27 wk old, sex NR), in situ, ultrasonic
			16.6 (0.48)		[65]	*n* = 10 (NZW, 2.8–3.2 kg, sex NR), ex situ
			16.64 (0.66)	15.12–18.56	[69]	*n* = 100 (mixed species, avg 2.4 kg, avg 41 mo, 21 F, 29 M), in situ, CT
			16.8	16.4–17.4	[63]	*n* = 5 (NZW, 14–22 wk old, M), ex situ
Vitreous volume, mL	1.84 (0.12)	1.59–2.22	1.06 (0.09)		[71]	*n* = 26 (DB, 2.0–2.5 kg, sex NR), ex situ
			1.15	0.72–1.50	[72]	Review of multiple studies
			1.4	1.3–1.6	[63]	*n* = 5 (NZW, 14–22 wk old, M), ex situ
			1.5 (0.1)	1.31–1.7	[13]	Review of multiple studies
			1.54 (0.14)		[73]	*n* = 12 (NZW, 3.0–3.5 kg, half M, half F), ex situ
			1.7	1.5–1.8	[74]	Review of two studies

n, number of animals; NZW, New Zealand White rabbits; DB, Dutch belted rabbits, NR; not reported, in situ; in vivo, ex situ; enucleated eye; F, female; M, male. In the present study, rabbits were female, weighed 2.9 to 4.0 kg, and were aged 23 to 27 weeks.

Additional methodological factors may also contribute to differences with previously reported values. This includes the use of in vivo MRI rather than ex vivo measurements following enucleation or dissection,^63^^,^^65^^–^^67^ as well as the effects of anesthesia during MRI acquisition, which has been associated with transient changes in ocular dimensions. Circadian variation may represent a further source of variability,^64^ although most experimental procedures in the present study were conducted in the morning to limit such effects. Differences in cohort composition may also contribute to variability between studies. All rabbits in this study were females. Whereas sex-related differences in ocular dimensions have been reported in juvenile rabbits, with male rabbits exhibiting significantly larger eyes than female rabbits up to 8 weeks of age,^68^ ocular size in adult rabbits appears to be more strongly associated with body weight than sex per se.^69^ As such, sex-related differences are unlikely to be a major determinant of the discrepancies observed here.

One objective of this study was to compare simulations based on vitreous geometries with different levels of anatomic detail, to determine which ocular measurements are required to reliably predict the influence of eye size and geometry on ocular half-life. To this end, three mathematical models were developed with increasing anatomic realism: a model based on optical AL alone, a second incorporating both AL and VD, and a third using reconstructed ocular geometries derived from MR images. Simulated half-life values were broadly consistent across vitreous models. Ocular biometry models based on AL alone produced similar predictions to those incorporating AL and VD (AL+VD; see Fig. 6). Including VD had minimal impact on the results, indicating that the simpler model provides sufficient generality and that measuring the VD is not essential.

In these models, ocular half-life showed a strong linear correlation with AL, closely matching the trend predicted by the MRI-based models (see Fig. 8). The MRI geometries, however, exhibited greater variability, with half-life values more closely associated with vitreous volume (see Fig. 7). Although the simpler models did not reproduce the individual half-life values obtained from the MRI geometries, where eyes of similar AL produced differing *t*_1/2_, they captured the overall trend. These findings suggest that the model-predicted ocular half-life is influenced by factors beyond AL and VD, whereas simpler models remain suitable for population-level predictions. Given the cost and limited accessibility of MRI, ocular biometry-based models provide a practical approach for estimating general IVT kinetics.

A second objective was to evaluate the influence of injection location on drug residence time in the computational models. Using the ocular biometry (AL) models, half-life was calculated for both a central and a more peripheral injection site, located approximately 9.5 mm and 3.9 mm from the edge of the ciliary body, respectively (Supplementary Fig. B3 of Supplementary S1.B). The half-life differed by approximately 0.6 days between these locations across the range of ALs (see Fig. 6). This variation was comparable in magnitude to the effect of eye size, suggesting that variability or uncertainty in injection location may obscure experimental correlations between ocular half-life and ocular dimensions.

The in vivo PK study was designed to generate experimental concentration-time data for the estimation of ocular half-lives in individual eyes. The goal was to evaluate whether the correlation between ocular half-life and eye size predicted by simulations could also be observed experimentally.

Based on the inclusion criteria, 20 eyes from the single-dose group and 28 eyes from the multiple-dose group had sufficient data for parameter estimation. Although parameters were identifiable for all eyes, several estimates had wide CIs (see Fig. 10a). Eyes with more frequent and later AH sampling yielded narrower CIs for the elimination rate constant (Supplementary Fig. F1 in Supplementary S1.F), consistent with established best practices showing that reliable estimation of elimination rates requires adequate sampling during the decay phase.^70^ The study protocol included additional sampling and a larger cohort; however, terminal samples were unusable, reducing both the number of eligible animals and the availability of late-phase data. Consequently, half-life estimates carried greater uncertainty. The decay model provided a good fit to the data (see Fig. 9), supporting the validity of the model structure described by Equation 3.

From a procedural standpoint, a single IVT injection was generally well-tolerated, whereas repeated IVT injections were associated with increased ocular inflammation, a consideration that may be relevant for the design and feasibility of similar longitudinal ocular PK studies.

Ocular half-life was then evaluated as a function of AL and vitreous volume (see Fig. 10). No correlation was observed in the full dataset (see Fig. 10a). However, a weak association emerged when restricting the analysis to estimates with narrower CIs (95% CI = < 1 day; see Fig. 10b), suggesting that estimation uncertainty, interindividual variability, and the limited range of eye sizes may have partly obscured an underlying relationship. Previous studies have proposed a relationship between ocular half-life and vitreal volume based on a spherical approximation of the vitreous chamber, and this framework has subsequently been used for interspecies comparisons based on differences in vitreal radius.^13^^,^^75^ To assess the applicability of this approximation in our dataset, we compared MRI-derived vitreous volumes with volumes predicted from AL using the same spherical model (Supplementary S1.E). The two approaches showed good qualitative agreement, although the spherical approximation consistently overestimated vitreous volume. In our results (see Fig. 10b), AL and vitreous volume showed similar correlations with ocular half-life (*R*^2^ = 0.15 vs *R*^2^ = 0.16, respectively). This is consistent with findings from our previous study,^15^ where a sensitivity analysis showed AL to have a greater influence on drug residence time than VD, suggesting that vitreal half-life may reasonably correlate with AL. Formal comparison of regression slopes further showed no significant difference between experimental and model-based relationships, indicating consistent prediction of a positive association between AL and ocular half-life (see Fig. 11). This agreement supports the robustness of the MFPT framework, which was developed independently of the PK data and without parameter fitting.

Previous studies reported ocular half-life estimates for ranibizumab in the range of 2.75 to 3.3 days (see Table 5), with a trend toward higher values in heavier animals. The present study used relatively large animals (2.9–4.0 kg body weight) and determined a population mean ocular half-life of 3.67  ±  0.54 days. This estimate is consistent with previous results, in particular with that reported by Shatz et al.^23^ with animals of comparable weight (3.3 days, 3.1–4.1 kg). Although the ocular PK studies listed in Table 5 did not report ocular size, these data are available from separate studies summarized in Table 4, which predominantly include smaller animals. Extrapolation of the linear regression derived from our PK data (see Fig. 10b) to the range of ALs reported in the literature (14.96 to 16.8 mm; see Table 4) yields a predicted ocular half-life of 2.77 to 3.45 days, consistent with previous results (see Table 5). Differences in study design and experimental methods may also have contributed to variability across studies.

**Table 5.** Reported Ocular Half-Life of Ranibizumab in Rabbits, With Corresponding Rabbit Weights and a Brief Description of the Study Methodology

Ocular Half-Life, d	Body Weight, kg	Age, Wk	Method	Reference
2.75	1.5–2.0	NR	Terminal sampling	[19]
2.81	1.5–1.8	NR	Imaging	[43]
2.88	1.7–2.0	NR	Terminal sampling	[20]
2.9	2.7–3.3	NR	Terminal sampling	[22]
3.3	3.1–4.1	17	Terminal sampling	[23]
3.67 ± 0.54	2.9–4.0	23–27	In vivo sampling	Present study

New Zealand White rabbits were used in all studies except those of Bakri et al.^20^ and Christoforidis et al.,^43^ which used Dutch-belted rabbits.

NR, not reported, age corresponds to age at initial dosing.

Nonlinear mixed-effects modeling was used to further characterize variability in the data. The analysis indicated that the initial volume parameter was not reliably identifiable at the individual level, as evidenced by high shrinkage and lack of support for a random-effects term, whereas the elimination rate constant was estimable with moderate precision (Supplementary S1.G). This is consistent with the profile likelihood results, where many initial volume estimates exhibited large confidence intervals (Supplementary Fig. F1 of Supplementary S1.F). Incorporating a hierarchical structure accounting for both eyes within each animal improved estimation of the elimination rate constant and reduced shrinkage, indicating shared animal-level information. Variance decomposition suggested two sources of variability in the elimination rate constant: between animals and between eyes within the same animal, with the latter smaller but non-negligible (Supplementary S1.G). The resulting population-level ocular half-life estimate (3.58 days) was consistent with that obtained using the primary analysis (3.67 days).

This study assumed that elimination rate constants estimated from AH samples serve as a proxy for vitreal half-life, consistent with flip-flop kinetics wherein slow transfer from the vitreous to downstream compartments governs the terminal elimination phase.^76^^,^^77^ This common approach is supported by empirical evidence: prior IVT ranibizumab studies in rabbits reported minimal differences (2%–3%) between half-life estimates derived from AH and vitreous samples.^20^^,^^22^ To evaluate whether target-mediated processes could confound these estimates, VEGF concentrations were assessed. In the present study, AH VEGF levels (0–400 pg/mL, or 0–10 pM) were lower than the reported binding affinity range for ranibizumab,^78^^–^^80^ indicating that target binding occurs in non-saturating conditions. Accordingly, target-mediated drug disposition is unlikely to have materially influenced the observed half-life, consistent with first-order elimination kinetics commonly reported in rabbit ocular PK studies.^14^^,^^20^^,^^22^

Although the computational models predicted a clear association between ocular half-life and AL, the filtered experimental data showed a weak correlation (*R*^2^ ≈ 0.15). Nevertheless, the slope of the experimental relationship did not differ significantly from those predicted by the models, supporting a consistent positive association. Furthermore, the positive lower bounds of the 95% CIs for the regression slopes provide additional evidence that the underlying relationship between eye size and ocular half-life is positive. Detecting this relationship in the present cohort was likely challenging due to the narrow range of eye sizes. AL varied by less than 1 mm across the central 90% of subjects and less than 1.5 mm across the central 95% (see Fig. 4). Applying the linear relationship for the filtered experimental results (see Fig. 10b) to the range of AL in the study cohort (16.35–18.10 mm; see Fig. 4) yields a maximal half-life difference across subjects of only 0.65 days (18% of the population mean), which is comparable to the variability predicted from differences in injection site (see Fig. 6). Together, these factors likely obscured the relationship between eye size and elimination in this rabbit cohort; it may be more apparent in populations with a broader distribution of ALs, such as an age-diverse sample.

In contrast, AL varies more widely in humans (approximately 21.6–26.7 mm).^15^ Extrapolating the linear relationship between AL and half-life derived in rabbits (see Fig. 10b) to the AL range reported in humans yields predicted ocular half-lives of 5.2 to 7.1 days, suggesting that AL alone could contribute approximately 2 days of variability across the population. This extrapolation is broadly consistent with clinical observations. For example, Avery et al.^12^ estimated the ranibizumab half-life at 5.8 ± 1.8 days (mean ± SD), indicating substantial interindividual variability that may arise, at least in part, from differences in AL, alongside other physiological and treatment-related factors. We do not consider these findings to be inconsistent with previous experimental studies reporting no correlation between intraocular drug concentration and eye size,^16^^–^^18^ as discussed further in the Supplementary S1.H. The influence of ocular geometry on half-life is predicted to be relatively modest and may therefore be difficult to detect in studies with limited cohort sizes, sparse sampling schedules, or other sources of uncertainty. Nevertheless, even modest differences in half-life, when compounded over a dosing interval, may have clinically meaningful implications. At a typical 4-week dosing interval,^81^ the extrapolated range of human half-lives would result in an approximately 9-day difference in the time required for drug concentrations to decline to a minimally efficacious level (25.1–34.3 days). Such differences could contribute to heterogeneity in ocular drug exposure among patients and may represent one factor underlying variability in treatment durability. Accordingly, further investigation of this relationship in humans is warranted, particularly given that AL can be readily measured using optical biometry.

## Conclusions

This study integrates MRI-derived ocular geometries, computational modeling, and in vivo PK data to investigate the influence of eye size on IVT drug elimination. A key contribution is the in vivo MRI-based characterization of ocular geometry in a cohort of adult rabbits, providing quantitative data on inter-eye variability that has been largely lacking in preclinical models. Model predictions indicated a relationship between AL and ocular half-life, which was not clearly detectable experimentally, likely due to the limited range of ocular dimensions in rabbits. Nonetheless, the experimental data were consistent with the overall trend predicted by the MFPT framework. The broader range of ALs observed in humans suggests that eye size may contribute more substantially to interindividual PK variability in clinical settings. We view investigation of this relationship in humans as a natural next step following the present study. If confirmed, this relationship could support the development of personalized IVT dosing strategies based on ocular biometry.

## Supplementary Material

Supplement 1

Supplement 2

Supplement 3
