# Supplementary material for: Individual-Eye Pharmacokinetics of Intravitreal Ranibizumab: Linking Ocular Geometry to Drug Retention Using MRI and Modeling
Source: Invest Ophthalmol Vis Sci. 2026 Oct 16;67(12):31. doi: 10.1167/iovs.67.12.31 (PMC13679778; doi:10.1167/iovs.67.12.31)
Supplement: Supplementary file 1 [file iovs-67-12-31_s001.pdf]

Supplementary Information for: Individual-Eye Pharmacokinetics of Intravitreal Ranibizumab: Linking Ocular Geometry to Drug Retention Using MRI and Modelling

Patricia Lamirande[1,7], Eamonn Gaffney[1], Michael Gertz[2], Basil Künnecke[3], Thomas Bielser[3], Andreas Bruns[3], Jessica Crawshaw[4], Uwe Wessels[5], Antonella Rampazzo[6], Philip Maini[1], Antonello Caruso[2].

[1] Wolfson Centre for Mathematical Biology, Mathematical Institute, Andrew Wiles Building, University of Oxford, Oxford, United Kingdom
[2] Pharmaceutical Sciences, Roche Innovation Center Basel, Roche Pharma Research and Early Development, Basel, Switzerland
[3] Neuroscience and Rare Diseases, Roche Innovation Center Basel, Roche Pharma Research and Early Development, Basel, Switzerland
[4] School of Mathematical Sciences, Queensland University of Technology, Brisbane, Australia
[5] Pharmaceutical Sciences, Roche Innovation Center Munich, Roche Pharma Research and Early Development, Penzberg, Germany
[6] Ophthalmology Section, Vetsuisse Faculty, University of Zurich, Zurich, Switzerland
[7] Department of Mathematics and Statistics and Sainte-Justine University Hospital Azrieli Research Centre, Université de Montréal, Montréal, Quebec, Canada

SI1 Table of Contents

A. Details on the experimental protocols 2
B. Details on the construction of the vitreous chamber geometries 6
Construction of the ocular biometry base model 6
Construction of the MRI models 8
Construction of the injection location points for comparison 10
C. Validation of the construction of the ocular geometries 12
Impact of the Gaussian filter 12
Impact of displacement magnitude in construction of boundary intersections 13
D. Validation of the profile likelihood assumptions for PK fitting 16
E. Imaging results and further analysis 17
F. Ranibizumab pharmacokinetic study results 21
G. Results of the nonlinear mixed-effects analysis 28

A. Details on the experimental protocols

The complete schedule implemented in the study is detailed in Figure A1, with the animal ID of the animals used in each group and sampling time, after replacements or changes in the planned study protocol.

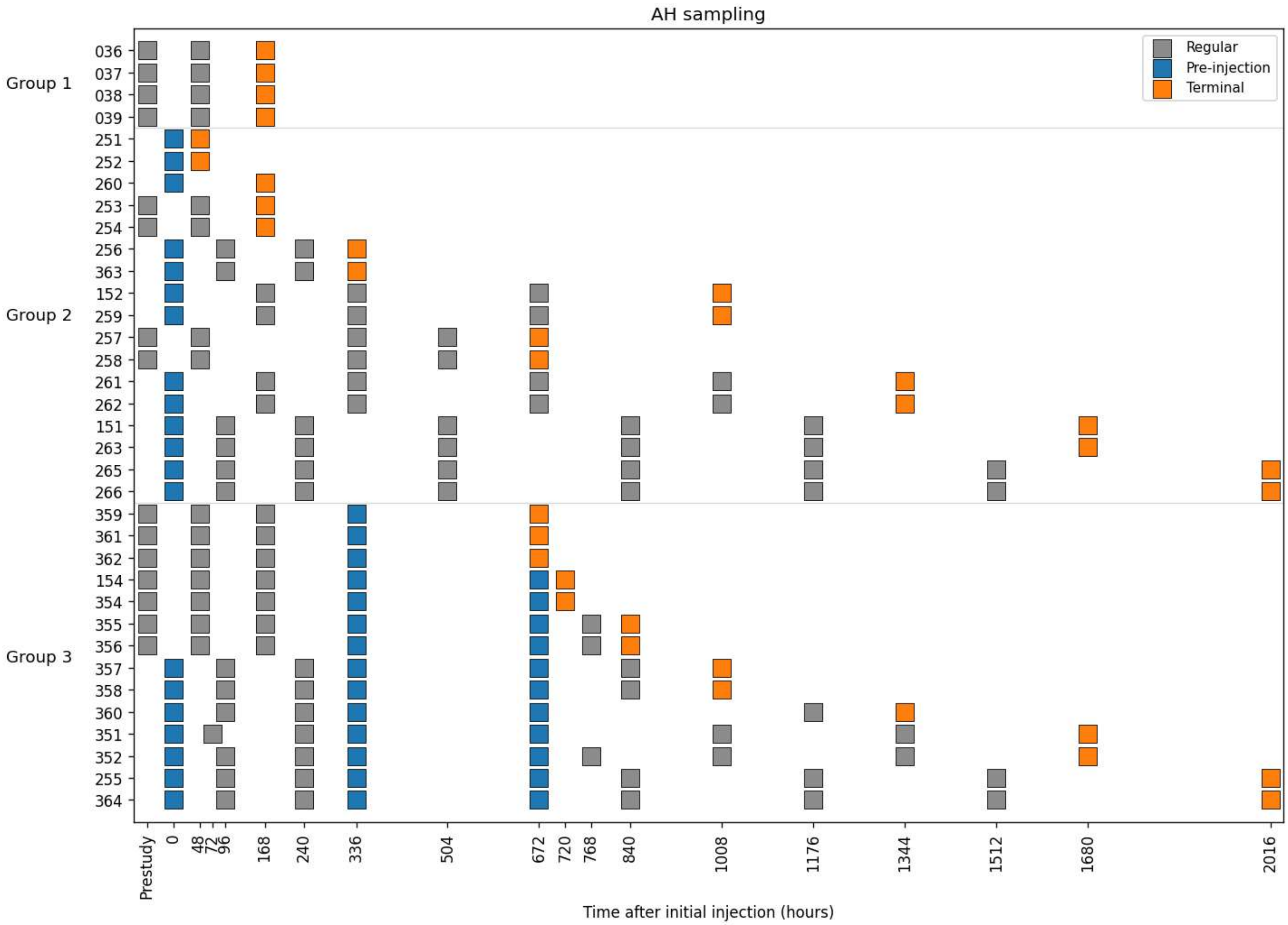

Figure A1. Schedule of aqueous humour (AH) sample in the three groups, with animal IDs listed in the vertical axis. Blue: samples taken prior to intravitreal dose, orange: post mortem samples, and grey: regular samples. Group 1: vehicle dose at 0 h; Group 2: single dose of ranibizumab at 0 h; Group 3: three doses of ranibizumab at 0, 336, 672 h. Both eyes were used in Groups 2 and 3 while, in Group 1, only the right eye was used for the injection and in vivo sampling, and both eyes were used for the post mortem samples.

Ultrasound ocular biometry

A-scan ocular biometry was performed using a Quantel Medical ABSolu ultrasound platform (Lumibird Medical, Cournon d'Auvergne, France). The system was equipped with a TP-02-las biometry probe, operating at 11 MHz and providing a nominal linear resolution of 0.1 mm. Individual alert animals were carefully restrained in a canvas "bunny snuggle" and one drop of 1.0% tetracaine was applied to each eye. Bilateral ultrasound assessments were performed in the contact mode starting with the left eye. After adding a drop of ultrasound gel to the tip of the ultrasound probe, the latter was placed perpendicular to the central corneal surface of the left eye. Acoustic feedback indicated the optimal placement of the probe and subsequently ten replicate measurements were obtained in rapid succession within two seconds. This procedure was performed three times per eye.

The acquired data were automatically evaluated by the manufacturer's built-in analysis algorithm. This analysis was based on the assumption of a phakic eye with a natural vitreous, and specific speeds of sound of 1532 m/s in the anterior chamber, 1641 m/s in the lens, and 1532 m/s in the vitreous, respectively. However, the assessment algorithm, which was tuned primarily for the size of human eyes, frequently failed to detect the posterior extent of the lens capsule, leading to incorrect or truncated readouts. Due to these flaws, the minimum reliably measurable change in size from baseline was 10%. This rendered the A-scan unsuitable for the current purpose and consequently, ultrasound biometry data were not further pursued.

Exclusion of terminal samples for parameter estimation

Inspection of the aqueous humour (AH) concentration-time data indicated that terminal samples were more variable than earlier time points and, in several cases, inconsistent with the expected decay profiles. Comparison with model-based predictions showed that terminal concentrations differed by a median of approximately 50%, with many samples exhibiting deviations greater than 70% and some extreme outliers exceeding 100% (Table A1). These discrepancies were not observed at earlier time points and were inconsistent with the monotonic decline seen in in vivo samples.

Terminal samples were collected ex vivo from enucleated eyes immediately following euthanasia (approximately 1–2 minutes between death and sampling), whereas all other samples were obtained in vivo from intact eyes (see Methods). In addition, terminal sampling involved removal of larger volumes of AH (typically 40–100 μl versus 40 μl in vivo), leading to the collapse of the anterior chamber and to vitreous prolapse.

Although the impact of such sampling conditions has not been systematically evaluated for AH pharmacokinetics following intravitreal antibody administration, post-mortem changes in ocular physiology may affect measured concentrations. Previous work has shown that aqueous humour composition is altered after death, with substantial increases in total protein levels and the appearance of plasma-derived proteins, consistent with disruption of the blood-aqueous barrier and increased compartmental mixing. These alterations are reported to begin immediately after death, although their magnitude has primarily been characterised over longer post-mortem intervals. Such changes, together with potential disruption of aqueous humour dynamics and intraocular pressure during ex vivo sampling, may influence the distribution of drug between ocular compartments and affect measured concentrations.

Given the high variability and frequent deviations observed in terminal samples, and their potential to bias estimation of elimination rate constants, these samples were excluded from parameter estimation.

Animal ID	Eye	Terminal time (days)	Measured ranibizumab (ng/ml)	Predicted ranibizumab (ng/ml)	Relative error (%)	Log-scale residuals
151	Left	70	0.3	<0.2	34.7	0.19
151	Right	70	0.6	<0.2	68.9	0.51
152	Left	42	10.4	10.5	0.9	0.00
152	Right	42	31.4	5.3	83.2	0.78
257	Left	28	639.3	240.8	62.3	0.42
257	Right	28	525.9	270.4	48.6	0.29
258	Left	28	637.3	262.0	58.9	0.39
258	Right	28	276.3	225.1	18.5	0.09
259	Left	42	15.4	32.8	113.3	-0.33
259	Right	42	7.5	5.2	30.1	0.16
261	Left	56	<0.2	0.7	261.5	-0.56
261	Right	56	0.7	0.6	15.7	0.07
262	Left	56	0.6	1.1	76.7	-0.25
262	Right	56	0.5	0.4	32.8	0.17
263	Left	70	0.3	<0.2	29.5	0.15
263	Right	70	2.7	<0.2	92.5	1.13
265	Left	84	<0.2	<0.2	0.0	0
265	Right	84	<0.2	<0.2	0.0	0
266	Left	84	<0.2	<0.2	0.0	0
266	Right	84	<0.2	<0.2	0.0	0
154	Left	30	2.3	9118.5	392866.0	-3.59
154	Right	30	<0.2	22532.6	11266206.4	-5.05
255	Left	84	3.3	0.7	77.7	0.65
255	Right	84	3.7	1.2	66.6	0.48
351	Left	70	19.8	13.0	34.5	0.18
351	Right	70	15.5	7.5	51.7	0.32
352	Left	70	18.6	7.8	58.2	0.38
352	Right	70	10.0	3.1	69.5	0.52
354	Left	30	45695.3	13540.6	70.4	0.53
354	Right	30	30501.5	30416.0	0.3	0.00
355	Left	35	11952.6	9169.9	23.3	0.12
355	Right	35	10191.0	8701.2	14.6	0.07
356	Left	35	19311.8	11169.2	42.2	0.24
356	Right	35	12943.1	11232.8	13.2	0.06
357	Left	42	4731.3	1880.3	60.3	0.40
357	Right	42	4152.2	2433.5	41.4	0.23
358	Left	42	5480.0	2240.3	59.1	0.39
358	Right	42	5245.1	3384.0	35.5	0.19
359	Left	28	11.5	2176.1	18894.2	-2.28
359	Right	28	31.9	2505.1	7760.6	-1.90
360	Left	56	186.0	354.3	90.5	-0.28
360	Right	56	134.7	138.9	3.2	-0.01
361	Left	28	<0.2	2740.6	1370188.9	-4.14
361	Right	28	<0.2	2391.5	1195651.7	-4.08
362	Left	28	6241.5	3225.4	48.3	0.29
362	Right	28	7336.1	2251.7	69.3	0.51
364	Left	84	2.8	0.7	76.6	0.63
364	Right	84	2.1	1.3	39.4	0.22

Table A1. Ranibizumab concentrations in aqueous humour at terminal sampling compared with model-based predictions, including relative error and log-scaled residuals. The lower limit of quantification (LLOQ; 0.2 ng/ml) was applied to model predictions. When either measured or predicted values were at the LLOQ, minimum relative error and residual values were reported (indicated in gray).

B. Details on the construction of the vitreous chamber geometries

Construction of the ocular biometry base model

The base model for the ocular biometry models, illustrated in Figure 2, was constructed using the average measurements from the MRI study, following the methodology introduced in our previous article and described below.[1]

The vitreous cavity was assumed to be an oblate ellipsoid, obtained by the rotation of an ellipse about its minor axis, yielding a symmetry around the optical axis of the eye. The semi-major axis of the vitreous ellipse, a, was defined as the vitreous radius, i.e. half of the vitreous diameter when measured on the axis perpendicular to the optical axis. The minor axis of the vitreous ellipse was defined as the difference between the full optical axial length, AL (measurement F in Figure 1), and the anterior chamber depth, ACD (measurement C in Figure 1), along with the section of the lens sitting outside the vitreous, identified as $l_T/4$, where l_T is the lens thickness. This value was determined from visual inspection of the MRI data from the imaging study, where approximately one quarter of the lens thickness was consistently observed to lie outside what would correspond to the ellipse representing the vitreous cavity. Hence, the semi-minor axis of the vitreous chamber, b, was set to

$$b = \frac{AL - (ACD + l_T/4)}{2}, \quad \text{(A.1)}$$

and its length is schematised in Figure 2. The lens protruding into the vitreous was also defined as an oblate ellipsoid, with the major axis corresponding to the lens diameter, l_D, and the minor axis to the lens thickness, l_T. Finally, the vitreous chamber was defined as the difference between the two ellipsoids. The base model was constructed using the mean measurements presented in Figure 4 for lens thickness, lens diameter, anterior chamber depth, vitreous diameter, and optical axial length. The plane geometry used in the axial rotation to define the 3D ocular biometry base model is illustrated in Figure 2, and the parameter values are listed in Table B1. In the ocular biometry models, to represent the variability of eye sizes, parameter b was varied using equation (A.1) as a function of the optical axial length measurement, AL, from the MRI study, noting that all other parameters (including ACD and l_T in equation (A.1)) were kept at their baseline.

Parameter	Description	Base model value (mm)
a	Semi-major axis of the vitreous cavity ellipse	8.79
b	Semi-minor axis of the vitreous cavity ellipse	6.25
l_D	Lens diameter	11.2
l_T	Lens thickness	7.59
h_{va}	Height of the vitreous-aqueous humour interface	1.10

Table B1. Parameter descriptions and values used for the construction of the baseline geometry for the vitreous ocular biometry model. The semi-axis b was obtained using equation (A.1), with the additional parameter values $AL = 16.9$ mm and $ACD = 2.50$ mm. The parameter h_{va} was defined to match the ratio of the surface area of the vitreous-aqueous humour interface with the MRI models developed in the next section (see text below for more details). The other parameters were directly derived from the average measurements from the MRI data study (Figure 4).

Three interfaces were defined on the surface of the vitreous geometries, representing the vitreous-lens interface, $\partial\Omega_{vl}$, vitreous-aqueous humour interface, $\partial\Omega_{va}$, and vitreous-retina interface, $\partial\Omega_{vr}$. Anatomically, the vitreous-retina interface corresponds to the surface covered by the inner limiting membrane and delimited by the ora serrata, while the vitreous-aqueous humour interface corresponds to the zonular fibres and space of Petit. Constructing the 3D geometry, the vitreous-lens interface was defined as the surface of the protruding lens. We defined the height of the vitreous-aqueous humour interface, h_{va}, to separate the remaining surface, representing the distance in the direction of the optical axis between the anterior limit of the vitreous and the ora serrata. We set $h_{va} = 1.1$ mm, so that the surface ratio of the vitreous-aqueous humour to the total surface in the base model was

$$\frac{S^*}{S} = \frac{\text{Surface area of } \partial\Omega_{va}}{\text{Total surface area } (\partial\Omega)} = 8.69\% \quad \text{(A.2)}$$

so as to correspond to the average ratio $S^*/S \approx 8.5\%$ in the MRI models developed in the next section.

Construction of the MRI models

Anatomically detailed vitreous models were constructed using the entire data from the rabbit MRI study. The main steps of the procedure, illustrated in Figure 3, are described below. After performing the MRI study, the segmentation of the main eye segments was implemented as described in the main text, and the corresponding masks were derived (steps **a** and **b** in Figure 3). A Gaussian filter was applied on the masks, performing a weighted average of surrounding pixels using the gaussian_filter function from the SciPy library in Python (Figure 3**c**). For the vitreous and the lens, the standard deviation for the Gaussian kernel was set to $\sigma_G = 4$, which yielded surfaces that were mostly smooth, with only minor irregularities caused by the limited resolution of the MRI. For the ciliary body, due to its thinner domain, a lower standard deviation was used $(\sigma_G = 2)$. While using a lower standard deviation for the Gaussian kernel avoided losing too much information on the region, it resulted in bumpy and uneven surfaces for the ciliary body. However, these irregularities were not transferred onto the final geometry, as the ciliary body was only used to define a boundary condition on the surface of the vitreous (as described later). The choice of the standard deviation for the Gaussian kernel and its impact on geometry construction is discussed and validated in SI.C.

Once the regions were smoothed, the meshes were created using the marching cube algorithm from the scikit-image library in Python, a 3D generalisation of isolines on topographical maps. With this method, each ensemble of masks was converted into a 2D surface mesh, resulting in a set of vertices and triangular faces. These sets were then converted into a mesh object using the Trimesh method from the trimesh library in Python, and then exported in stl format. Each mesh was then imported in COMSOL and converted into a COMSOL geometry mphbin file for further use (Figure 3**d**). At this stage, minor smoothing and simplifications were applied to the mesh, for example by joining COMSOL geometry entities, to ensure each geometry was formed of only one object.

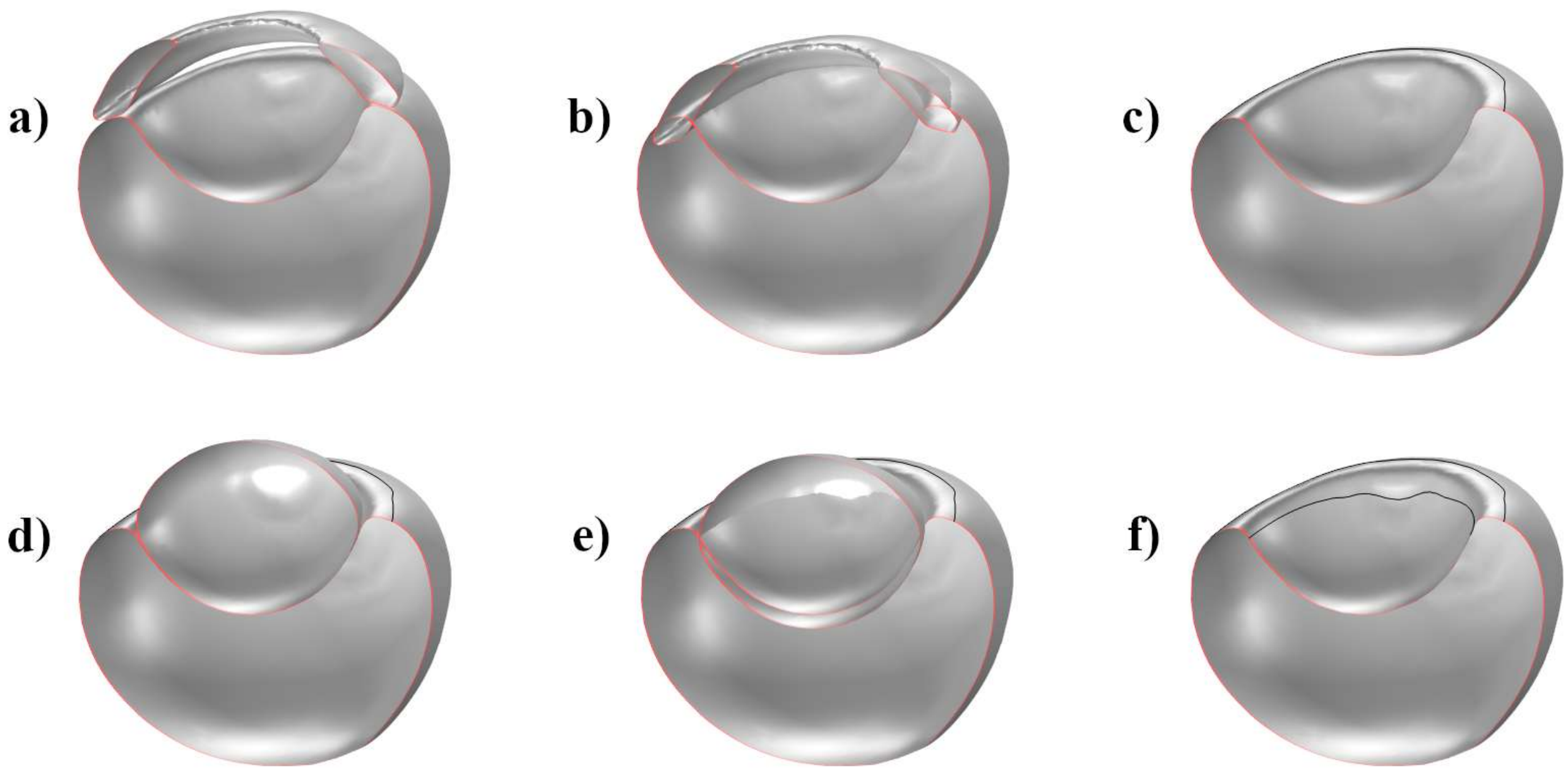

Figure B1. A schematic describing the construction of the surface interfaces for the geometry in Figure 3**e**. In **a)**, the ciliary body and vitreous geometries are imported. In **b**, the ciliary body is moved ≈ 1.0mm towards the vitreous, and only the outer intersection is kept in **c)**. The lens geometry is then imported in **d)**, and in **e)** is moved ≈ 0.6mm towards the vitreous. In **f)**, the intersection of the two objects remains.

Next, the geometry of the vitreous chamber with its three boundary conditions was constructed, using the lens and ciliary body to create the intersections, equivalent to the boundaries defined in the ocular biometry model in Figure 2. The main steps are schematised in Figure B1. The lens and the ciliary body did not completely overlap with the vitreous chamber (as can be seen in Figure B1**a**), which could be a result of the MRI precision and of the Gaussian filter that was applied. Therefore, the two structures had to be manually shifted towards the vitreous body to create a well-defined intersection. The vitreous-lens interface was defined as the intersection of the lens with the vitreous, applying a translation of 0.60–0.62 mm down the z-axis (corresponding to the sagittal axis of the reference frame) for the lens. The vitreous-aqueous humour interface was defined as the region delimited by the vitreous-lens interface, and by the outer intersection of the ciliary body with the vitreous, after a translation of 1.00–1.05 mm of the ciliary body down the z-axis. The vitreous-retina interface was defined as the remaining surface on the vitreous chamber, delimited by the vitreous-aqueous humour boundary. The displacements were kept within small ranges (instead of a fixed value) to circumvent any internal geometry

errors that sometimes occurred due to inconsistencies in COMSOL's intersection operations, where small adjustments could help resolve unexpected issues. The influence of the displacement choices on modelling results is assessed in SI.C. A representation of the obtained geometry is illustrated in Figure 3**e**.

Finally, a 3D mesh was generated in COMSOL for the volume and surface of the vitreous geometry (Figure 3**f**) and was used when solving the MFPT equation given in the main text. The refinement of the mesh was validated by confirming that the results obtained with the generated mesh did not differ by more than 0.2% from the results obtained by an extremely fine mesh. The results were consistent across a subset of four geometries (out of the 78 geometries constructed).

Construction of the injection location points for comparison

In all three models, based on previous work[1], a plausible injection location was modelled midway between the lens and retina along the visual axis. This point, referred to as the midpoint of the vitreous depth, allowed direct comparison of simulation results across eyes and models. In the ocular biometry (AL) and ocular biometry (AL+VD) models, its position was set on the visual axis (the axis of symmetry) and constructed using the half distance of b, as defined in equation (A.1) (see Figure B3). In the MRI models, the optical axis was first defined using the centres of gravity (COGs) of the vitreous, lens, and anterior chamber that were previously constructed. An example of this is shown in Figure B2**a**, where the optical axis is illustrated against the vitreous, lens, and anterior chamber surfaces for the left eye of subject 038. The midpoint on the vitreous depth was then defined as the central point between the intersection of the optical axis and the vitreous surface, as illustrated in Figure B2**b–d**.

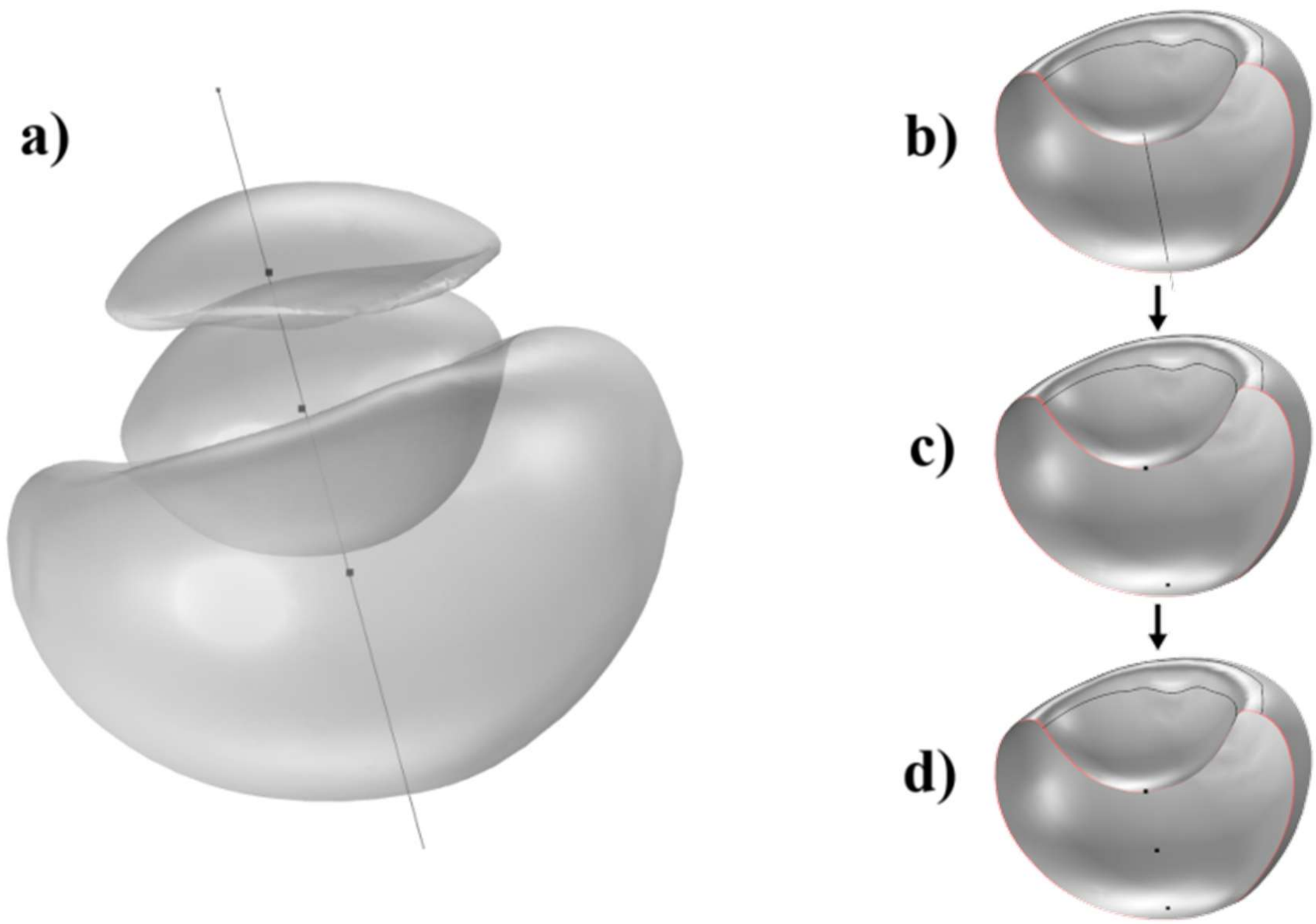

Figure B2. Construction of the vitreous depth midpoint for the left eye of subject 038. Left: Optical axis (line) defined as the best fit line across the centres of gravity of the anterior chamber, lens, and vitreous (points), as described in the text. Right: Construction of the optical axis (line in **b**), intersection between the optical axis and the vitreous surface (points in **c**), and midpoint on the vitreous depth (middle point in **d**).

An additional injection location was defined in the ocular biometry (AL) models. This point, referred to as the side point, represented a more anterior injection used to minimise the risk of lens injury in species with smaller eyes, and was defined closer to the needle entry point in the vitreous. It was positioned along the major axis of the vitreous cavity ellipse, where the vitreous diameter is the widest and perpendicular to the optical axis, at a distance of $2a/3$ from the optical axis (with a depicted in Figure 2). Both injection points are depicted in Figure B3 in the base geometry of the ocular biometry (AL) models.

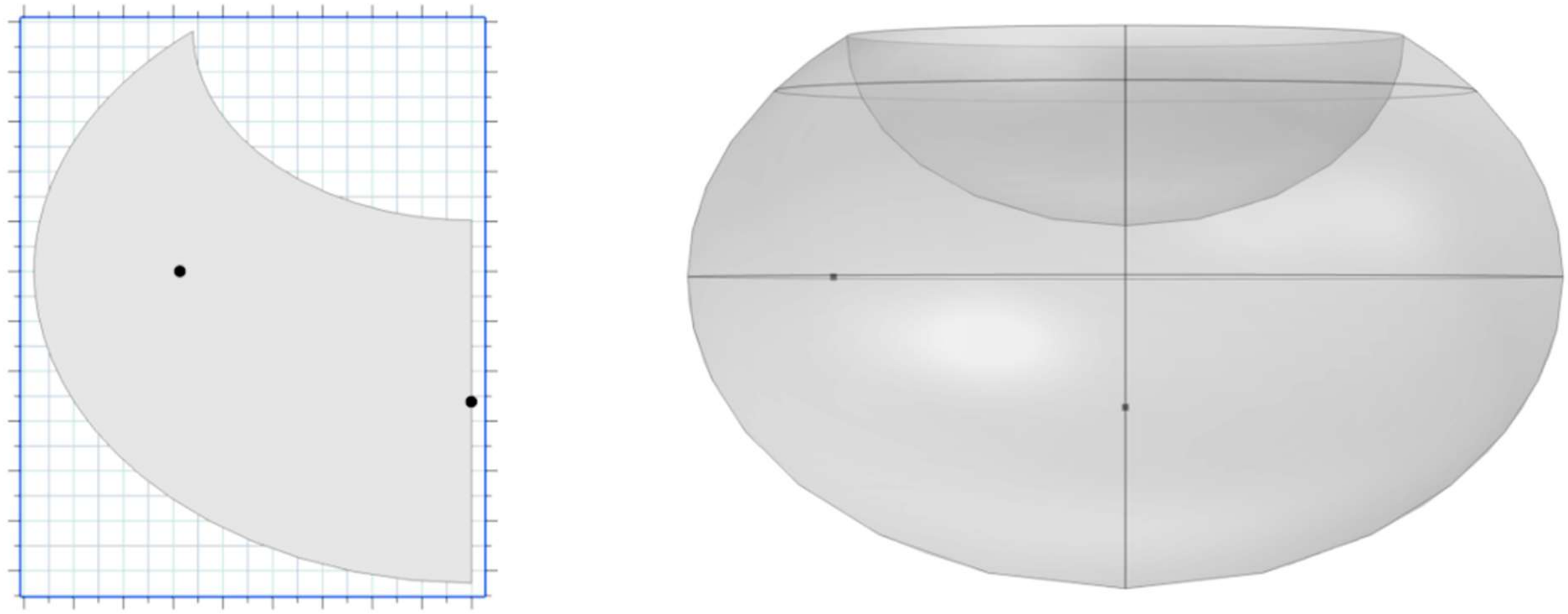

Figure B3. Plane geometry (left) and 3D model (right) for the base ocular biometry vitreous chamber geometry, with the points of interest. The point on the optical axis is denoted the vitreous depth midpoint, and the other point is the side point.

C. Validation of the construction of the ocular geometries

Impact of the Gaussian filter

The impact of the Gaussian filter and the standard deviation for the Gaussian kernel, σ_G, on the construction of the geometry was assessed by comparing the volume of the regions before and after smoothing. The reference volume (before smoothing) was defined as the volume of the respective segments reported in the MRI study, while the volume of the constructed segment after smoothing was measured in COMSOL. As this process had to be implemented manually for each eye segment and each subject, the comparison was performed only on a subset of 10 eyes of the population, using the left eyes of the subjects 036–040, 151–154, 251–258.

Overall, the vitreous volume of the constructed geometries was between 3.2 – 3.9% smaller than the volume measured directly from the MRI, and the lens volume between 7.1 – 8.3% smaller. This validated the choice $\sigma_G = 4$ for the vitreous and the lens regions, as it allowed the construction of smooth surfaces with only small variations in the total volume. For the ciliary body, the volume of the constructed geometries after smoothing was, on average, 17% smaller than the reported volume using MRI, with a difference up to 32% smaller.

While a smaller σ_G would better preserve the true volume after the Gaussian filter, it would also lead to a coarser and grainier mesh, making the geometry construction less straightforward (see Figure C1 for a comparison of a constructed geometry with $\sigma_G = 0.5$ and $\sigma_G = 2$). This difficulty was probably due to the ciliary body's thickness being close to the MRI spatial resolution limits, making it difficult to extract a smooth object from the data. Hence, $\sigma_G = 2$ was set as a compromise between representativeness of the original object and smoothness of the created geometry (to avoid errors in implementing the intersection of objects in COMSOL).

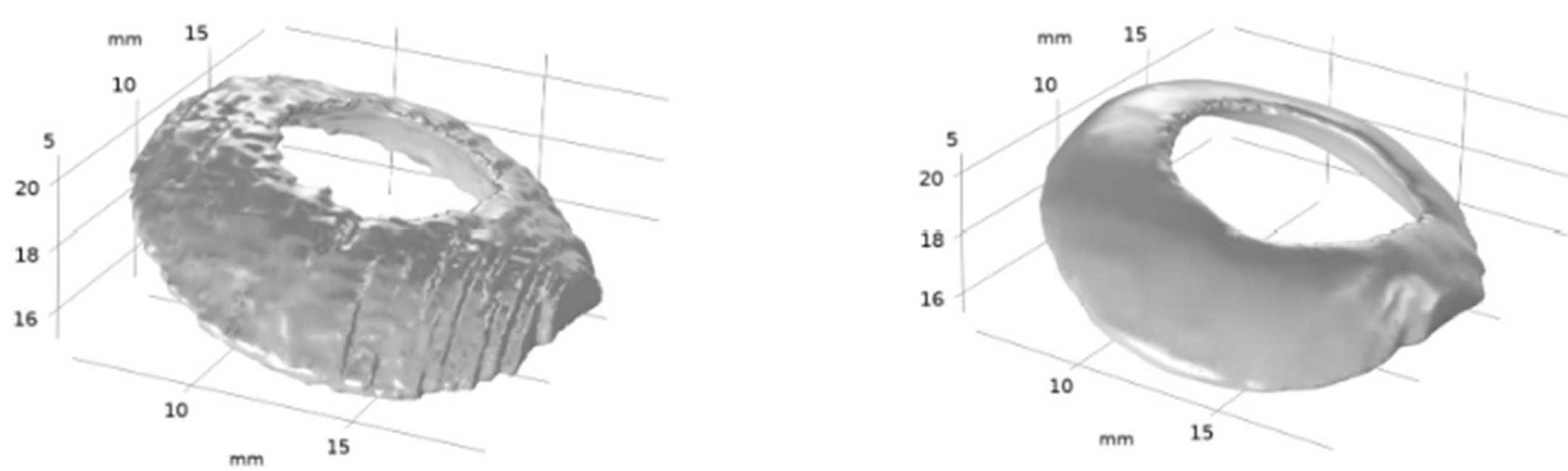

(a) $\sigma_G = 0.5$ (b) $\sigma_G = 2$

Figure C1. Ciliary body geometry constructed after applying a Gaussian filter with $\sigma_G = 0.5$ (left) and $\sigma_G = 2$ (right), for the left eye of subject 038. The geometry on the left is deemed too coarse, and the geometry on the right retained for the modelling.

Impact of displacement magnitude in construction of boundary intersections

As described previously, the different boundary conditions were defined by the intersection of the lens and the ciliary body after a displacement to create an intersection with the vitreous geometry. The impact of the displacement value on the results was assessed by comparing the effect of a displacement change on the resulting MFPT, for diffusing molecules starting at the midpoint on the vitreous depth. This analysis was carried out for two MRI geometries, the left eyes of subjects 038 and 251. Similar results were obtained

for both, supporting the assumption that we can expect similar impacts throughout the MRI geometries.

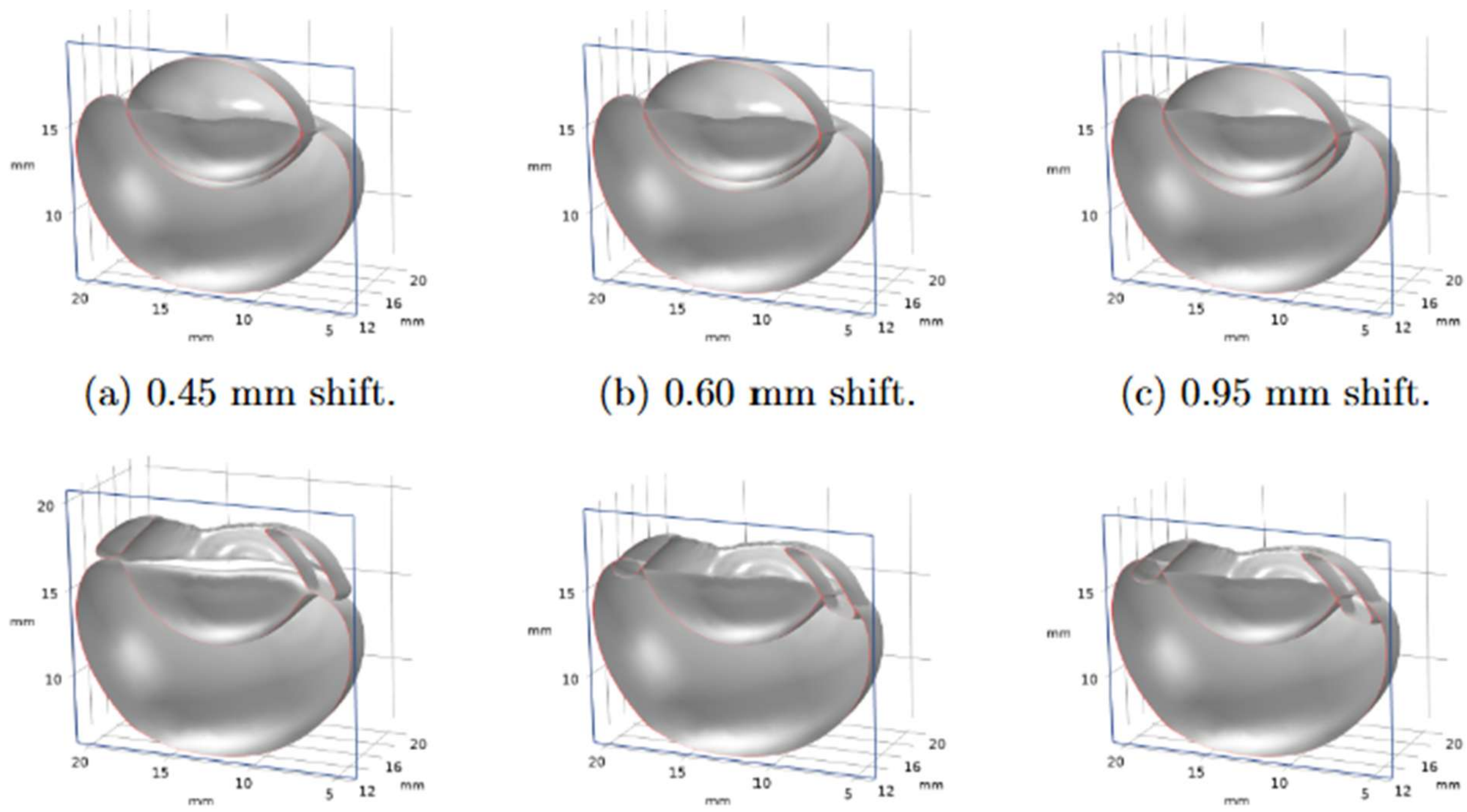

(a) 0.45 mm shift. (b) 0.60 mm shift. (c) 0.95 mm shift.

(d) No shift. (e) 1.05 mm shift. (f) 1.35 mm shift.

Figure C2. Minimum, baseline, and maximum displacement of lens (top row) and ciliary body (bottom row) down the z-axis onto the vitreous for the left eye of subject 038, creating the vitreous-lens, vitreous-aqueous humour, and vitreous-retina interfaces.

For the same vitreous chamber, different versions of the geometry were constructed by varying the lens displacement between 0.45 – 0.95 mm, effectively changing the location of the vitreous-lens boundary condition (increasing and decreasing the vitreous-aqueous humour interface surface area). The minimum and maximum lens displacements are illustrated in Figure C2 for subject 038, along with the displacement value used when constructing the MRI geometries (0.60 – 0.62 mm). A similar process was implemented for the displacement of the ciliary body, with displacements between 1.05 – 1.35 mm, where the minimum corresponds to the displacement used when constructing the MRI geometries (which varied between 1.00 – 1.05 mm). The minimum and maximum ciliary body displacements are illustrated in Figure C2 for subject 038, along with the segments without shift (where the ciliary body is just over the vitreous, with limited contact). The displacement

of the ciliary body was constrained due to the thinness of the ciliary body geometry and the need for it to intersect the vitreous in all places for a correct geometry to be created, which did not typically happen with displacements of less than 1.00 mm.

The MFPT was then obtained for each variant of the vitreous geometries for an injection at the midpoint on the vitreous depth, by solving equation (1) and boundary conditions (2) with the parameter values defined in the Methodology. The results are shown in Figure C3. The MFPT results did not vary much due to variations in the lens displacement. More specifically, there was only a difference of 0.6% between the minimum and maximum MFPT across all lens displacements for subject 038, and a difference of 1.4% for subject 251. Therefore, the magnitude of the lens displacement in the geometry construction had minimal impact on the MFPT results, making the choice to shift the lens by 0.60 – 0.62 mm as good as any.

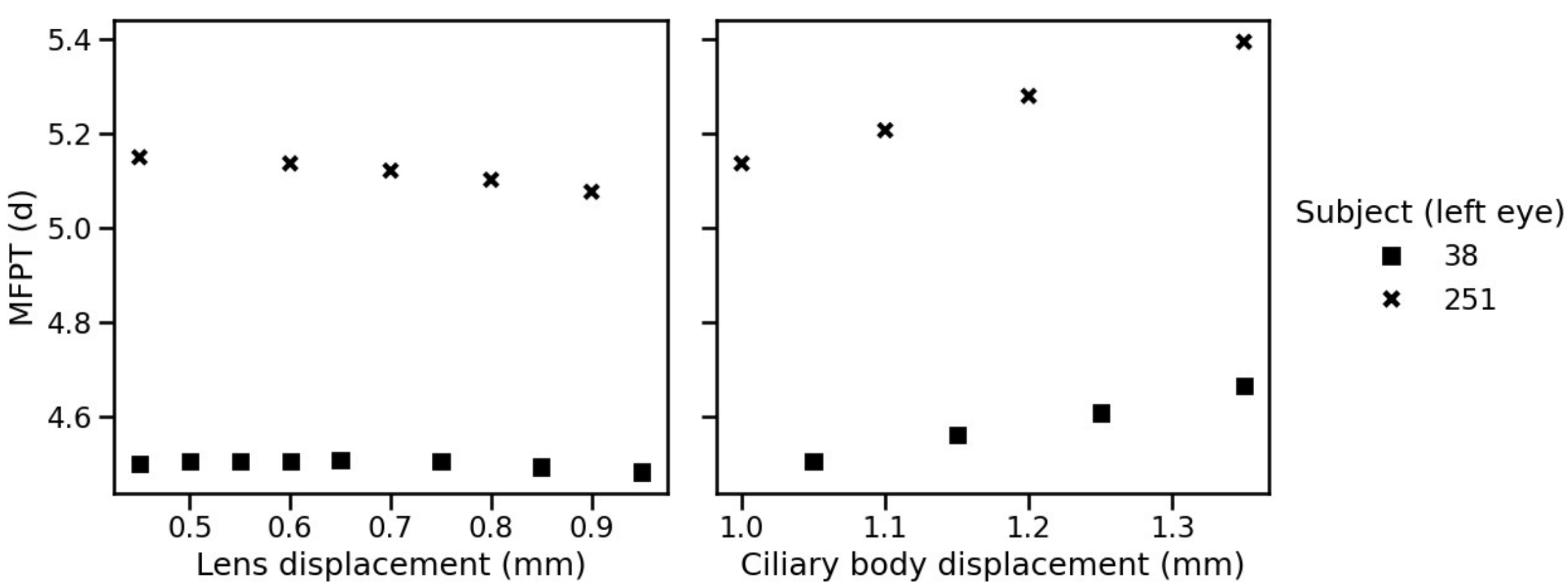

Figure C3. Mean first passage time (MFPT, days) from the midpoint on the vitreous depth, for varying lens and ciliary body displacements during the geometry construction, in the left eyes of subjects 038 and 251 from the MRI data.

For the ciliary body, there was a difference of 3.5% and 5.0% for the MFPT across the ciliary body displacement values for subjects 038 and 251 respectively, with longer MFPT for larger displacements. In Figure C2, we see that for larger displacements of the ciliary body (from 1.05 mm to 1.35 mm), the vitreous-aqueous humour interface becomes narrower, as the ciliary body is wider at its base, and narrows towards the anterior section.

Therefore, a smaller displacement would represent the anatomical interface more accurately (see the no shift panel in Figure C2). After a computational investigation, we found that the smallest displacement we can use to create the intersection that allows a complete closure of the gap between the vitreous and ciliary body for all points in COMSOL was in the range 1.00 – 1.05 mm, as used in the model to build the interfaces for the boundary conditions.

D. Validation of the profile likelihood assumptions for PK fitting

With the independence of the maximum likelihood estimate (MLE) values from the choice of the standard deviation in the inference framework, we set $\sigma = 0.1$ without loss of generality to obtain the MLE for V_0 and λ. These MLE values were then used to evaluate the residuals, by taking the difference between the model-predicted log concentrations (with parameters of equation (3) corresponding to their MLE values) and the experimental log concentrations. The log-scaled residuals were centred around zero with a sample standard deviation of $\sigma = 0.26$.

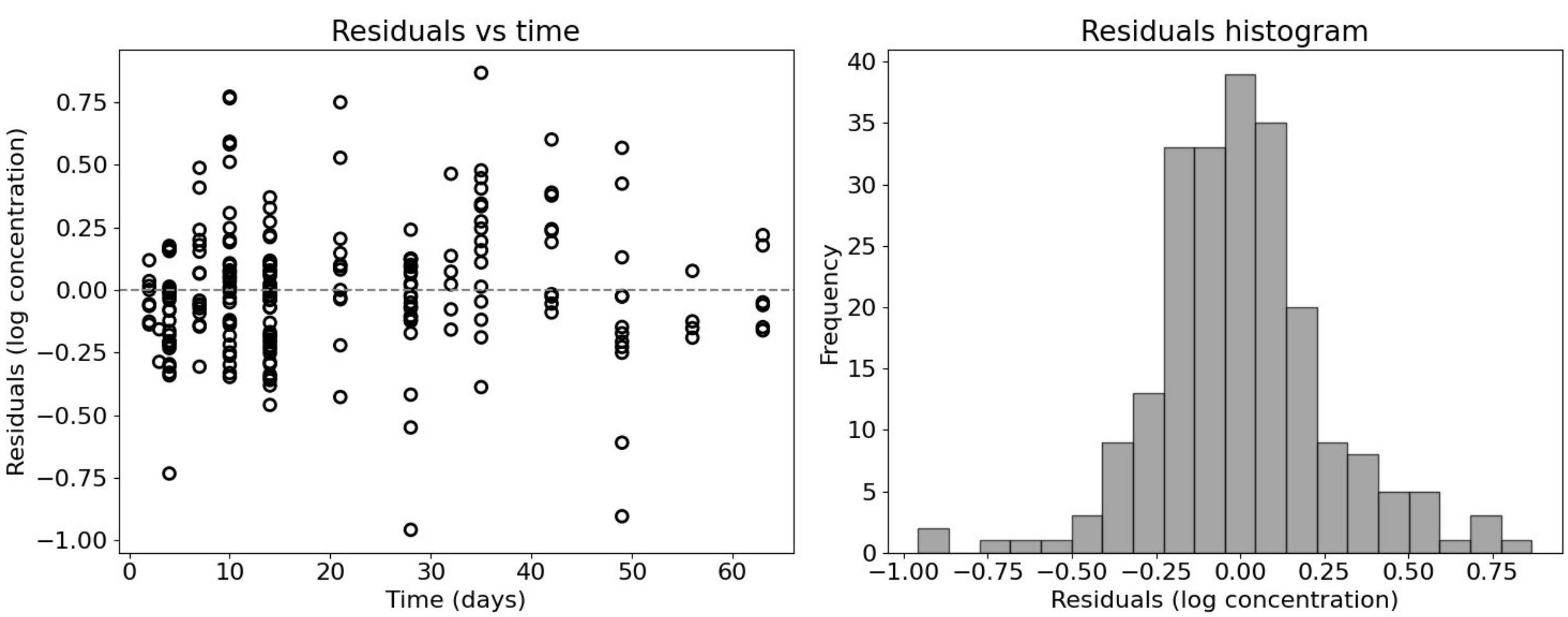

Figure D1. Left-hand side: distribution of the residuals for each concentration measurement as a function of time across all animals and eyes. Right-hand side: histogram of the distribution of residuals for all animals and eyes, where frequency corresponds to the number of observations within each interval.

Note that the distribution of the residuals on the left-hand side of Figure D1 shows no systematic variation with time, which would be consistent with the homoscedasticity assumption, a requirement when applying the profile likelihood framework. To formally assess this, White's test for non-linear models was applied to the residuals, using the het_white function from the statsmodels package in Python.[2] The statistical test confirmed that the residuals were equally scattered over time at the 95% confidence level.

On the right-hand side of Figure D1, the frequency distribution of the residuals resembles a normal distribution centred around zero. To formally test for normality, the Kolmogorov-Smirnov normality test was implemented in Python, using the kstest function from the stats package.[3,4] The distribution of the residuals was compared to a normal distribution with mean zero and standard deviation $\sigma = 0.26$, corresponding to the sample statistics of the residuals, and passed the normality test at a 95% confidence level. Therefore, for a standard deviation of $\sigma = 0.26$, the data met the normality and homoscedasticity conditions required for the assumption $ln\,(y(t)) \sim N(ln\,(C(t)), \sigma^2)$, which was the foundation of the profile likelihood approach presented in the main text.

E. Imaging results and further analysis

The individual results for the axial length and vitreous volumes for each subject eye are reported in Figure E1, where the results are shown for each subject with increasing axial length. The correlation between optical axial length and vitreous volume is shown in Figure E2. In the right hand-side of Figure E2, the relationship between the vitreous volume and the predicted vitreous volume using the spherical approximation of the vitreous chamber (taking the optical axial length (AL) as the diameter of the spherical vitreous chamber):

$$V = \frac{4\,\pi(AL/2)^3}{3}$$

is illustrated.

The distribution of the rabbit eye measurements was analysed to identify potential outliers. As a reference, we first examined the distribution of human eye dimensions to determine what type of distribution (e.g. normal or log-normal) could be expected. To assess the distribution of the human eye dimensions, measurements of axial length and vitreous volume summarised in our previous work[1] were used, based on the data from experimental studies[5-7]. Quantile-quantile plots comparing the distributions of axial length and vitreous volume with normal distributions (using the sample mean and standard deviation) are shown in Figure E3, demonstrating good agreement with normality. The normality was further confirmed by a Kolmogorov-Smirnov test at the 95% confidence level.

Assuming that rabbit eye dimensions would likely exhibit a distribution comparable to human eyes, the distributions of the axial length and vitreous volume measurements were compared to a normal distribution, with sample mean and standard deviation, in quantile-quantile plots in Figure E3. The observations mostly follow the normal distribution. We performed a Kolmogorov–Smirnov test for normality on each measurement, comparing the data to a normal distribution with sample mean and standard deviation estimated from the dataset, and both measurements did not lead to a rejection of normality at the 0.05 significance level. Although the rabbit eye dimensions for axial length and vitreous volume followed a normal distribution overall, some outliers were apparent for longer axial lengths and larger vitreous volumes. The most extreme deviations corresponded to the left and right eyes of animal 152, which were markedly larger than the other eyes and fell outside of the central 95% of the measurements for the axial length and vitreous volume. Only one more observation was outside of the central 95% of the measurements, and corresponded to the smaller eye of animal 039, which was part of the control group. Because the eyes of animal 152 deviated substantially from the distribution, their pharmacokinetic results were excluded from the analysis of general trends. Results were still computed for this subject, but not included in size-based comparisons to avoid skewing the overall trend.

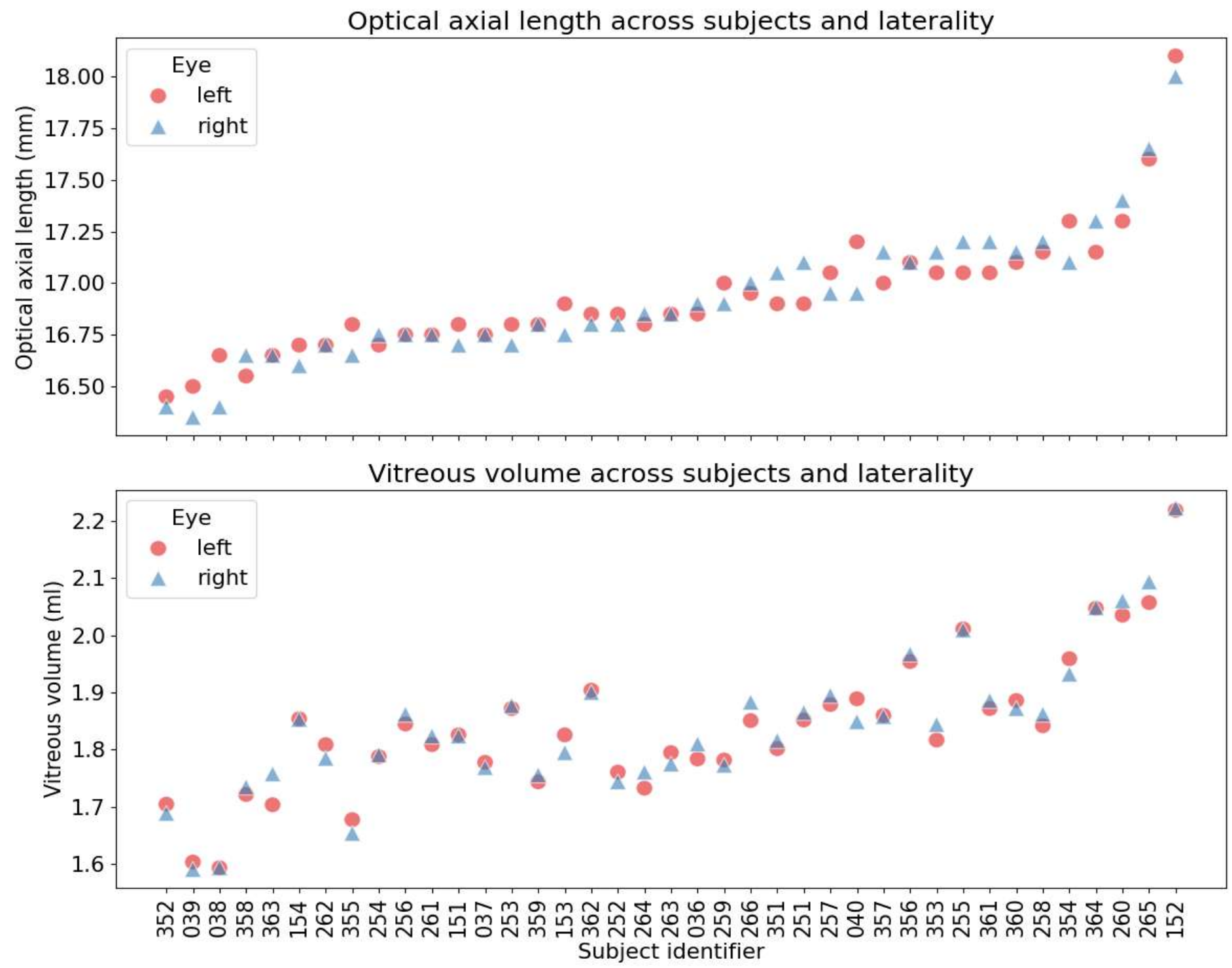

Figure E1. Inter-individual and intra-individual variability in optical axial length (top) and vitreous volume (bottom). Measurements are plotted for each rabbit and for every eye, with subjects ordered according to increasing optical axial length (average of left and right eye).

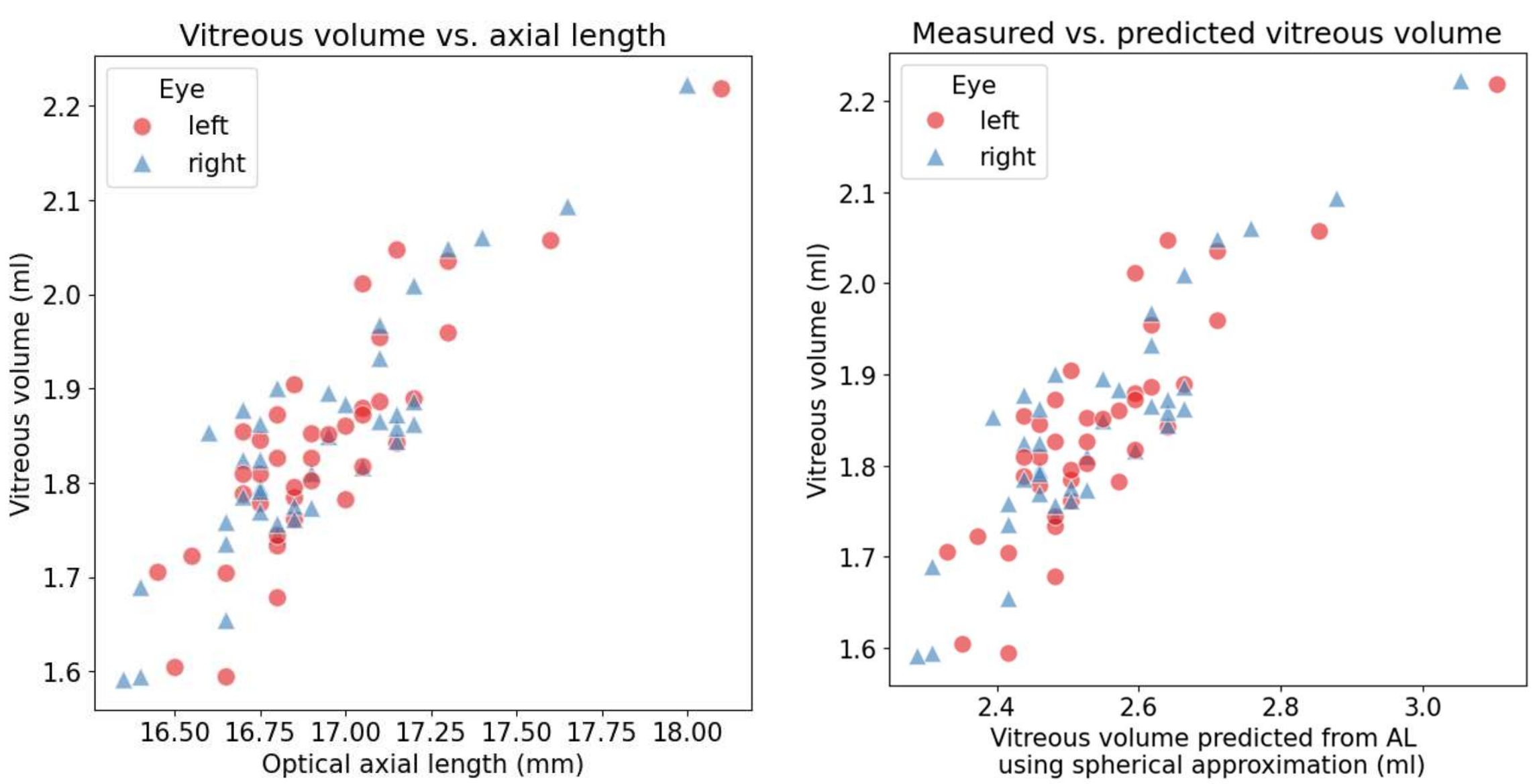

Figure E2. Left: MRI-derived vitreous volume and axial length measurements for each eye of all study subjects. Right: Measured vitreous volume plotted against volumes predicted under a spherical approximation, assuming the axial length corresponds to the diameter of the vitreous chamber.

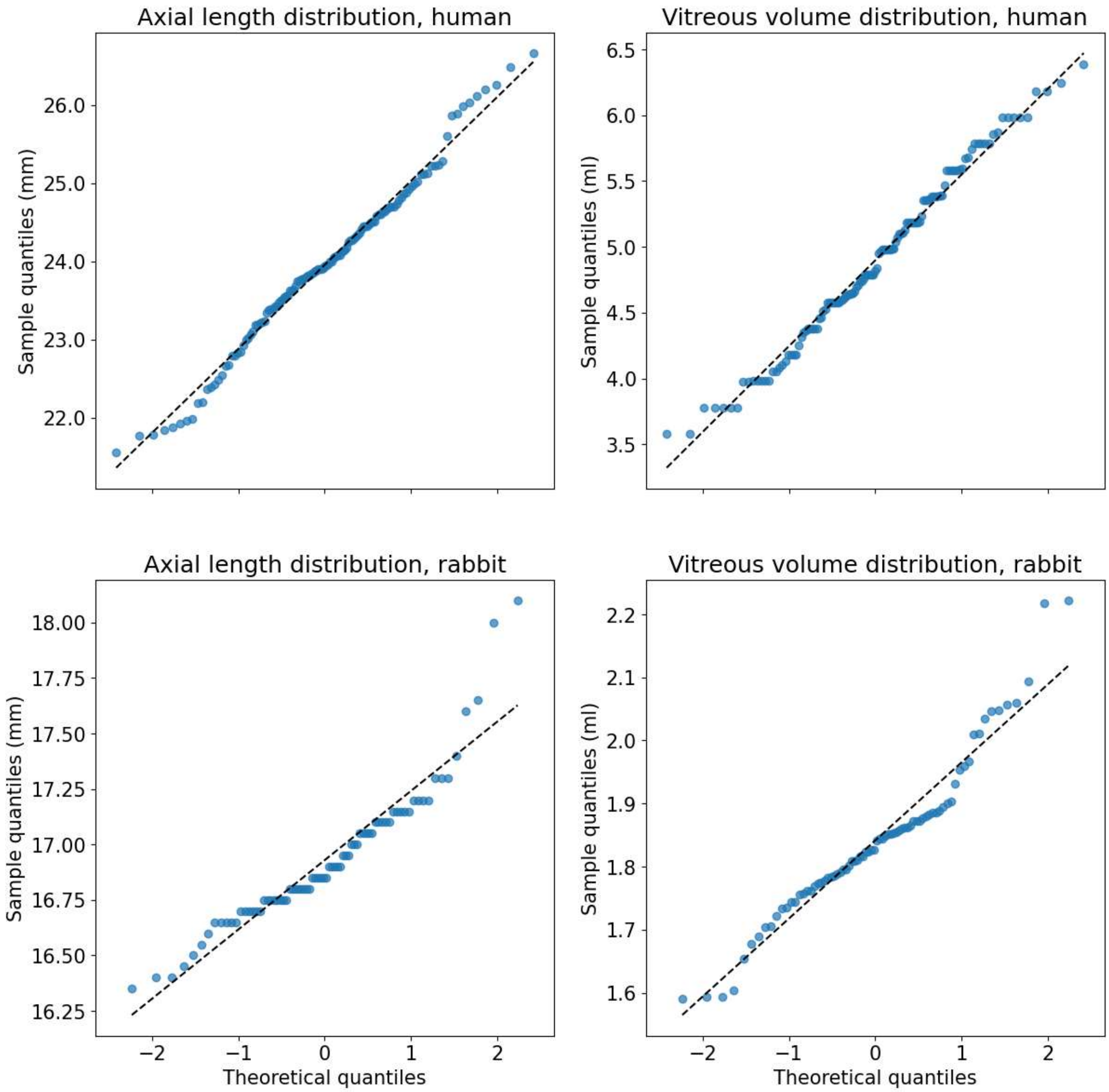

Figure E3. Quantile-quantile plot comparing literature measurements of axial length (left) and vitreous volume (right) measurements of human (top row) and rabbit (bottom row) eyes to normal distributions with sample mean and standard deviation. The points are observations ordered by quantile, with the corresponding measurement on the y-axis, while the dashed line represents perfect agreement with the normal distribution. The theoretical quantiles on the x-axis refer to the quantile values calculated from the normal distribution.

F. Ranibizumab pharmacokinetic study results

Parameters V_0 and λ were found to be practically identifiable for all eyes, with their MLE values and 95% confidence intervals shown in Figure F1 for each subject and eye. There is a wide variability in the length of the 95% confidence intervals across eyes, which can be explained due to the varying number of observations available for fitting. Narrower confidence intervals for λ values were found for subjects with numerous aqueous humour samples over a longer time after injection. The fitted equation (3), with the parameters corresponding to the MLE values for each eye, are compared to the experimental observations for the single-dose group in linear and log-linear plots in Figures F2 and F3 respectively, and for the multi-dose group in linear and log-linear plots in Figures F4 and F5 respectively.

The results from the MFPT modelling in the MRI vitreous chambers are compared to the experimental $t_{1/2}$ in Figure F6, where the results are plotted as a function of axial length and vitreous volume. As stated in the main text, the ocular half-life was obtained from the MFPT results using equation (6). Only the simulation results for the eyes with PK data are shown in Figure F6, with the simulated ocular half-life from the MRI modelling represented as blue triangles, and excludes subject 152. We see that the inter-individual fluctuation of ocular half-life from the MRI modelling results was less pronounced than for the estimates obtained from the PK data, with the former MLEs varying within a range of 3 – 4.2 days, while the latter MLEs varied within a range of 2.8 – 4.8 days.

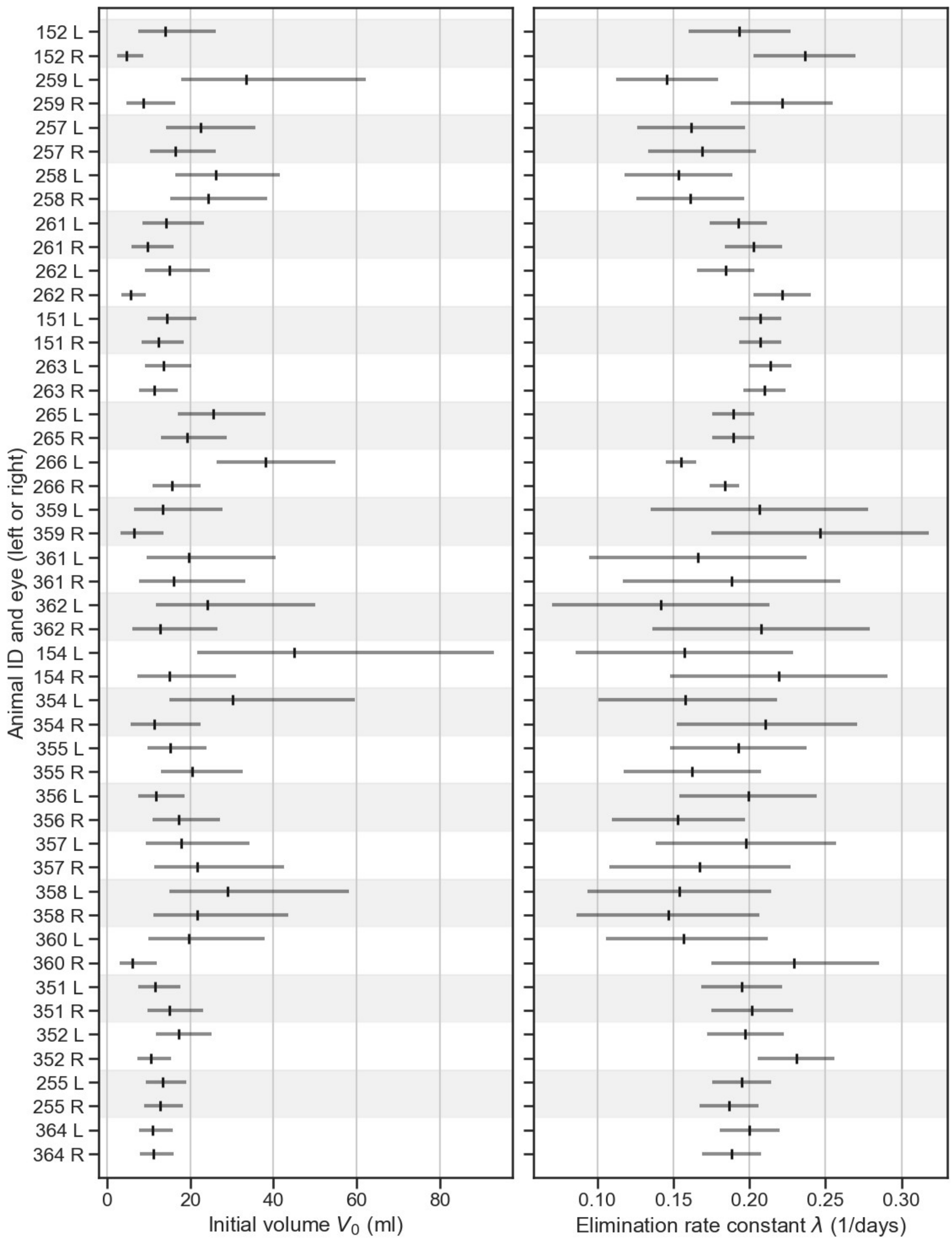

Figure F1. Maximum likelihood estimate (points) and 95% confidence interval (horizontal lines) for the initial volume, V_0, and the elimination rate constant, λ, for each eye and each subject (listed on the y-axis), estimated by fitting equation (3) to the pharmacokinetics data. The rows are shaded by animal subject for better visualisation, and the animals are listed by group and number of samples (following order in Figure A1).

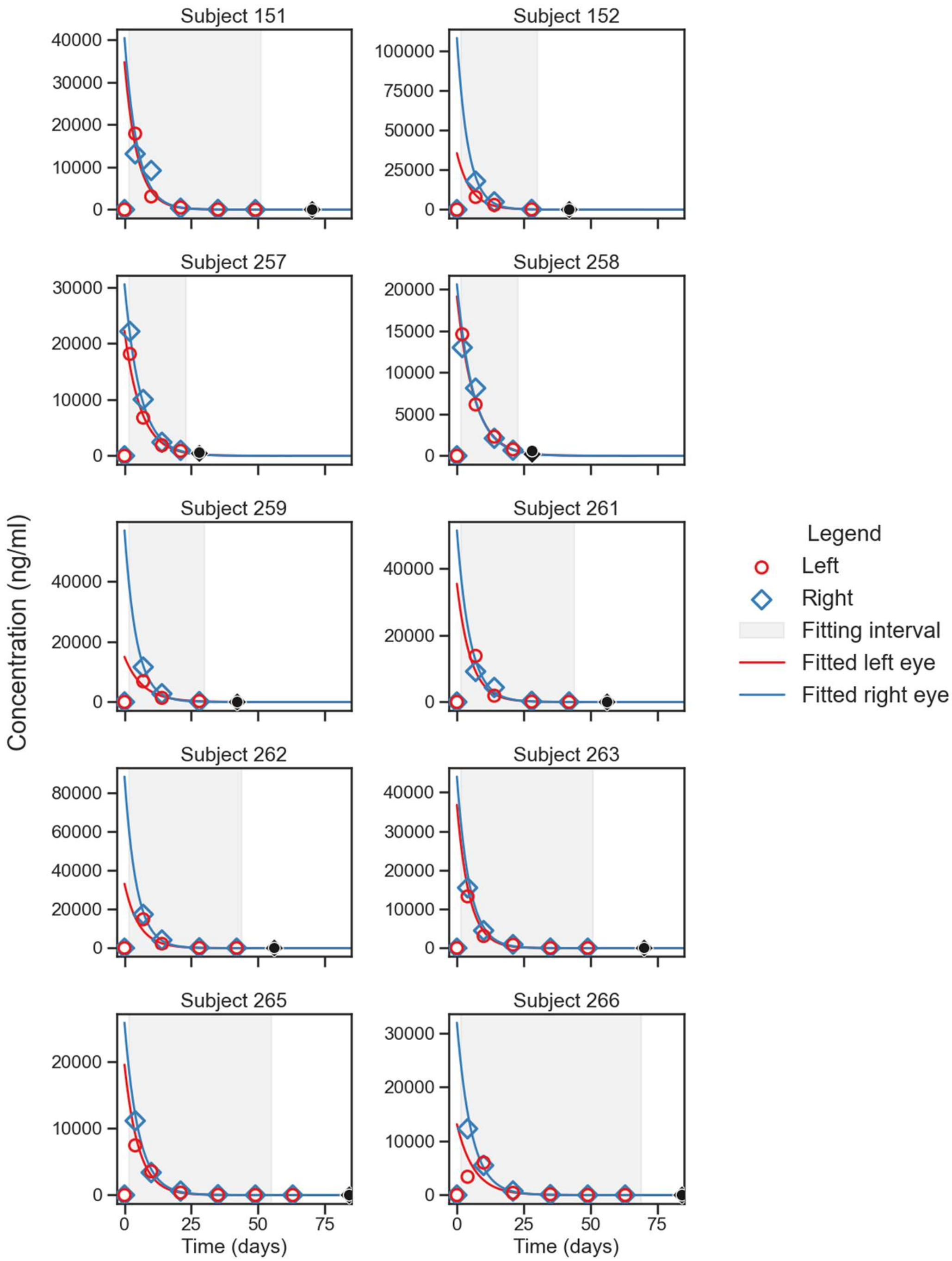

Figure F2. Concentration measurements of ranibizumab in aqueous humour samples over time for the left (dot) and right (cross) eye of each animal in the single dose group, along with the fitted model of equation (3) evaluated with the MLE parameter values. The grey region identifies the points included in the fitting, and excludes the concentration measurement on Day 0 (pre-dose). The measurements below the lower limit of quantification (LLQ) are plotted at the LLQ, but were excluded from the fitting procedure. The black dots and crosses identify the samples taken at termination time, which were also excluded from the fitting process.

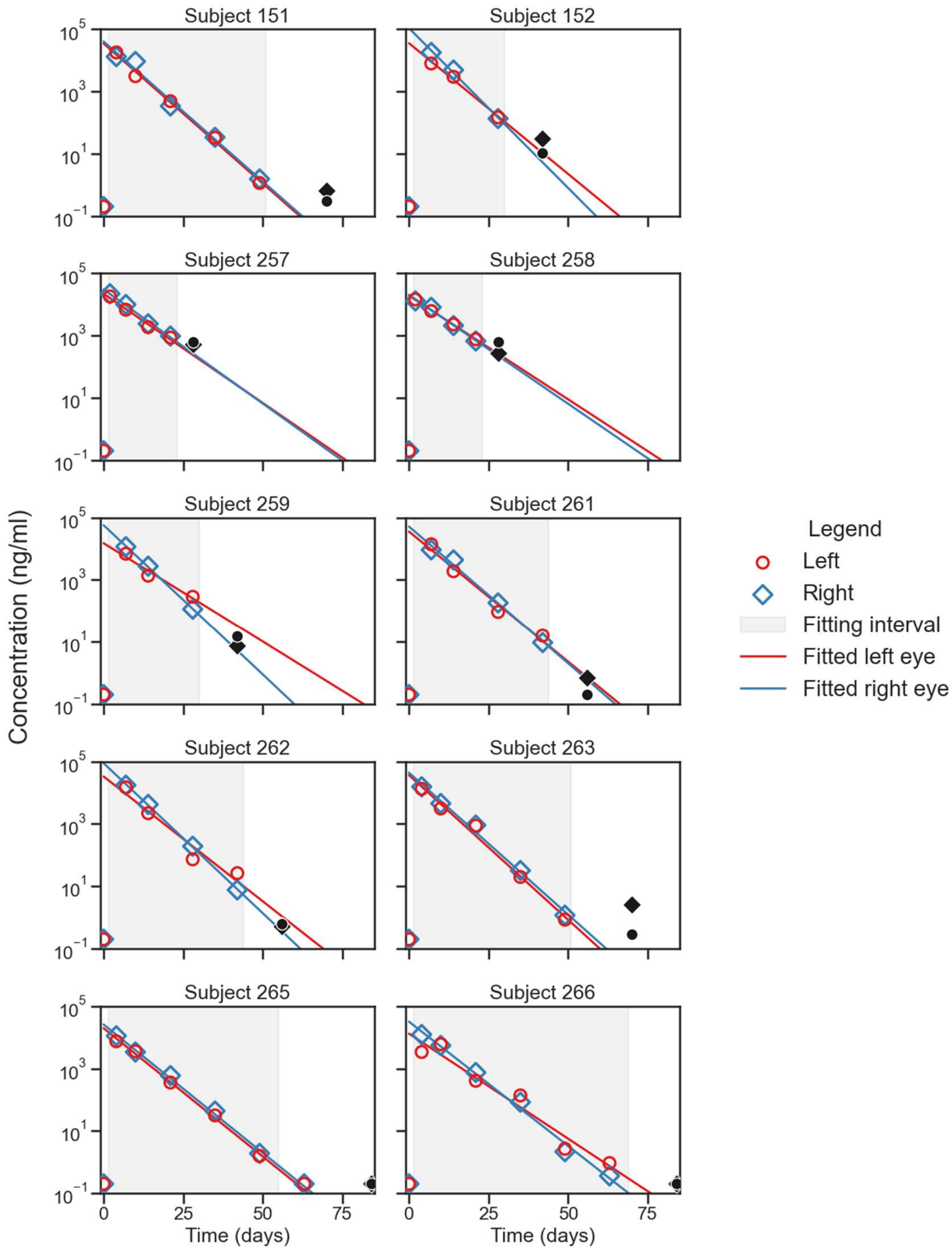

Figure F3. Semi-log scaled plots of the concentration measurements of ranibizumab in aqueous humour samples over time for the left (dot) and right (cross) eye of each animal in the single dose group, along with the fitted model of equation (3) evaluated with the MLE parameter values. The grey region identifies the points included in the fitting, and excludes the concentration measurement on Day 0 (pre-dose). The black dots and crosses identify the samples taken at termination time, which were also excluded from the fitting process.

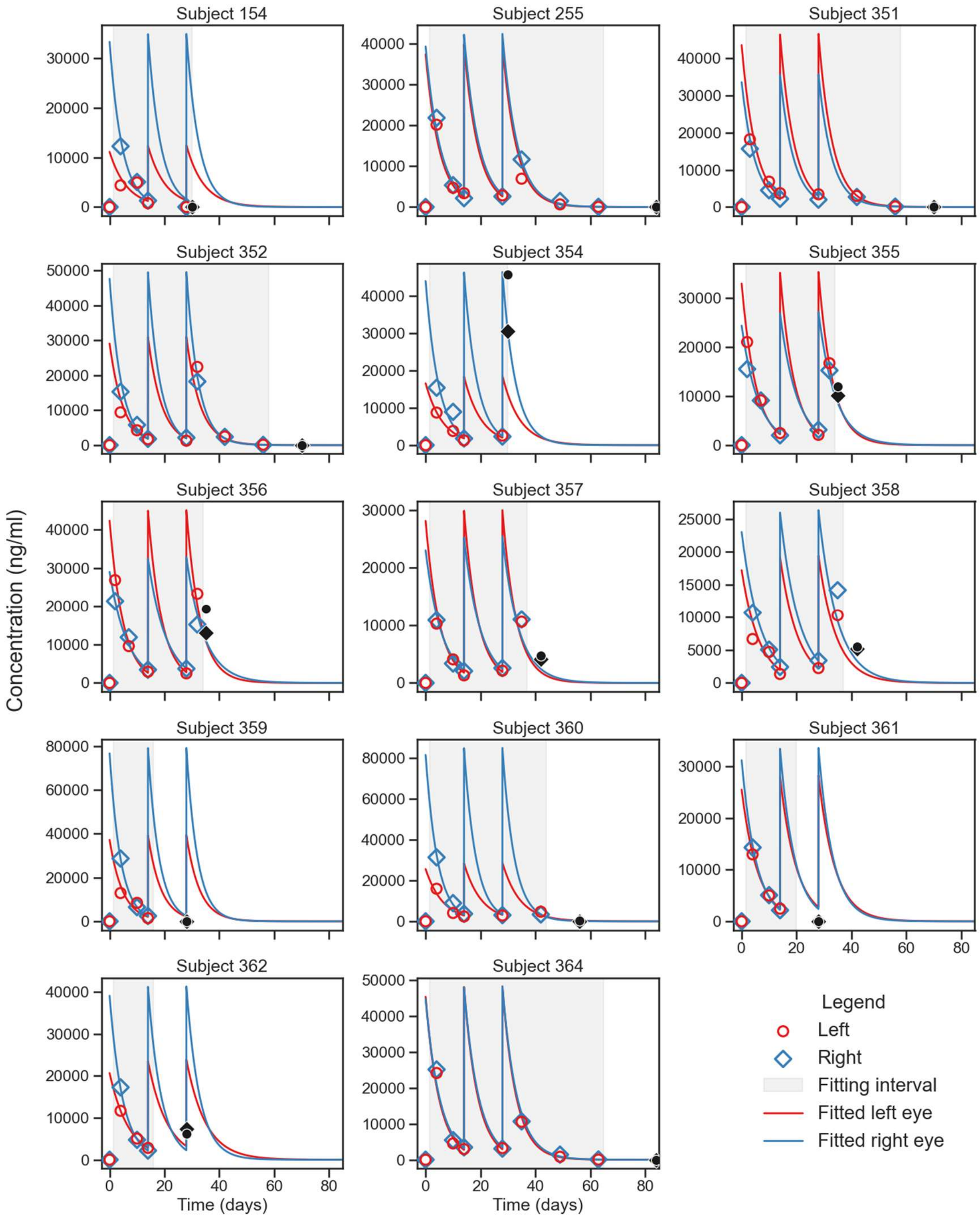

Figure F4. Concentration measurements of ranibizumab in aqueous humour samples over time for the left (dot) and right (cross) eye of each animal in the multi-dose group, along with the fitted model of equation (3) evaluated with the MLE parameter values. The grey region identifies the points included in the fitting, and excludes the concentration measurement on Day 0 (pre-dose). The measurements below the lower limit of quantification (LLQ) are plotted at the LLQ, but were excluded from the fitting procedure. The black dots and crosses identify the samples taken at termination time, which were also excluded from the fitting process.

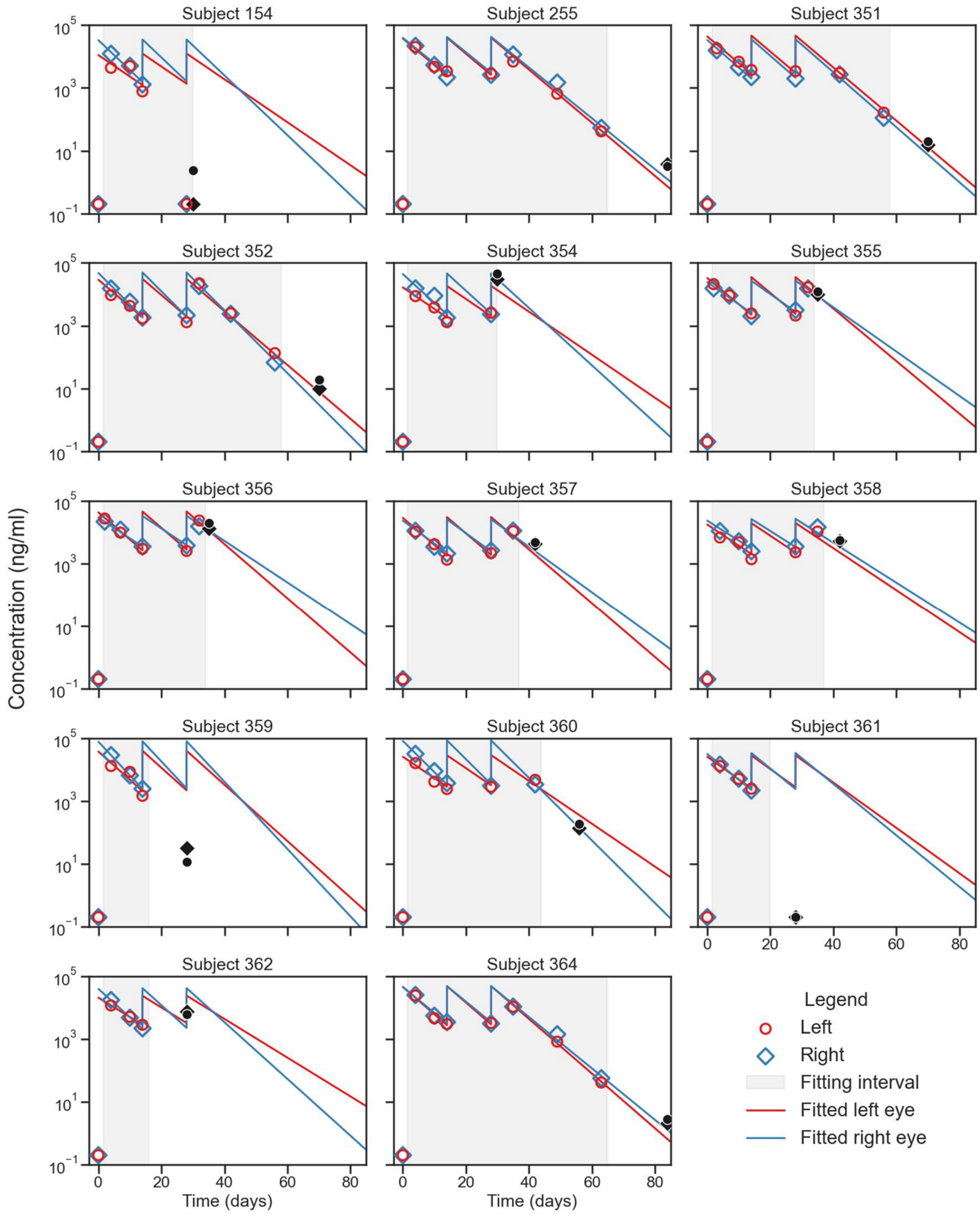

Figure F5. Semi-log scaled plots of the concentration measurements of ranibizumab in aqueous humour samples over time for the left (dot) and right (cross) eye of each animal in the multi-dose group, along with the fitted model of equation (3) evaluated with the MLE parameter values. The grey region identifies the points included in the fitting, and excludes the concentration measurement on Day 0 (pre-dose). The black dots and crosses identify the samples taken at termination time, which were also excluded from the fitting process.

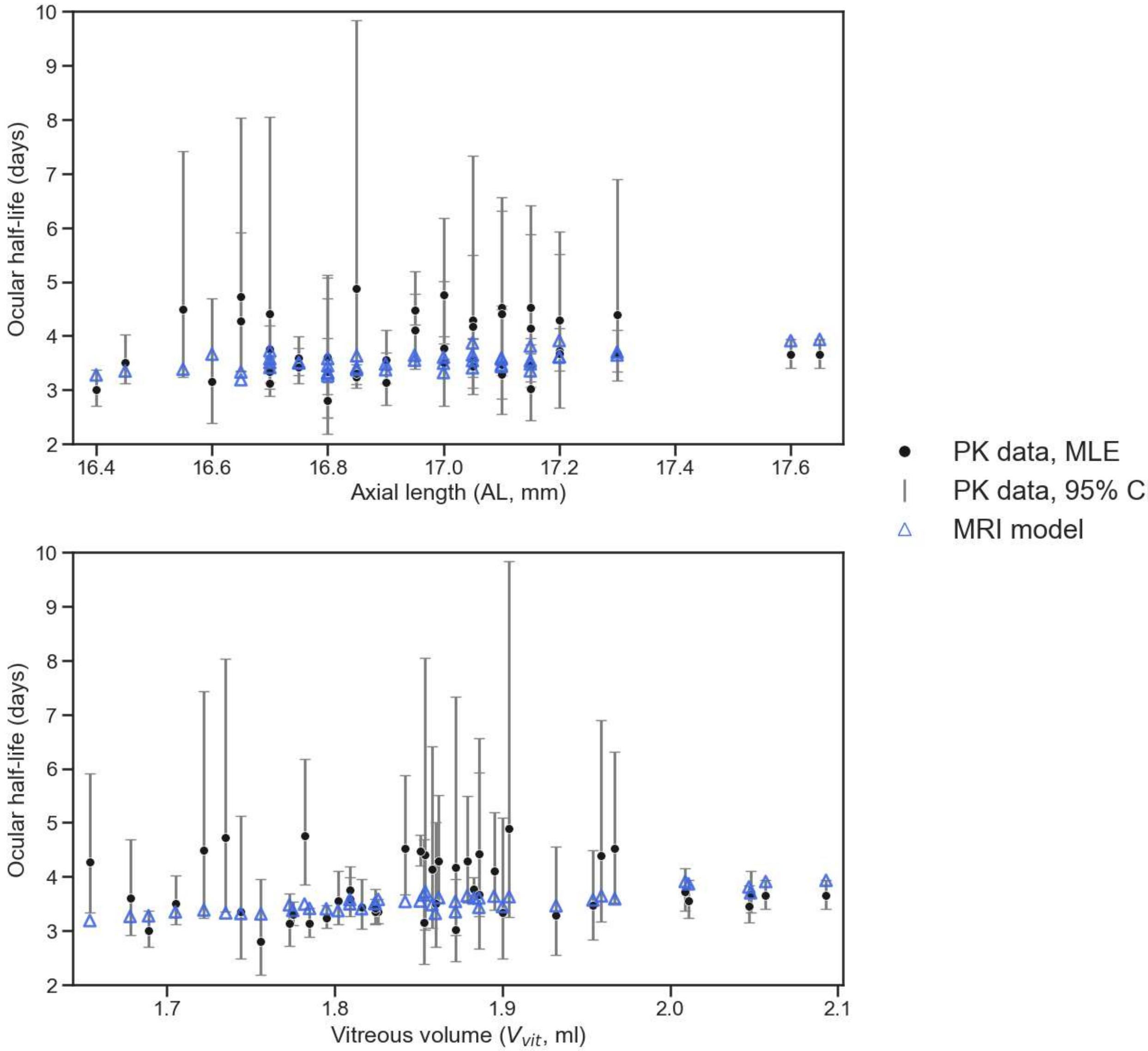

Figure F6. Maximum likelihood estimates (points) and 95% confidence intervals (bars) for the ocular half-life, $t_{1/2}$, from the pharmacokinetics study, and the corresponding estimates from the MRI models using computational simulations (blue triangles).

G. Results of the nonlinear mixed-effects analysis

Pharmacokinetic data were modelled using a one-compartment model with instantaneous (IV) bolus administration and first-order elimination, implemented in Monolix (version 2024R1; Lixoft SAS, Antony, France)[8]. The structural model is

$$C(t) = \sum_m \frac{D_m}{V} \exp\left(-k(t - t_{D,m})\right),$$

where D_m and $t_{D,m}$ denotes the m-th bolus dose and its administration time, k the first-order elimination rate constant, and V the apparent volume of distribution. The dose and administration time follows the description in the Methods section of the main text. Population parameters were parameterised log-normally. Inter-individual variability (IIV) was modelled using exponential random effects. Random effects were included on k but not on V. Inclusion of IIV on V resulted in high shrinkage and poor identifiability when IIV on k was simultaneously estimated; therefore, IIV on V was removed from the final model.

Inter-occasion variability (IOV) was included on k, with each eye of an individual treated as a separate occasion, allowing separation of inter-individual and intra-individual variability components.

Parameter estimation used the stochastic approximation expectation–maximization (SAEM) algorithm (exploratory phase 2500 iterations; smoothing phase 500 iterations). Initial population estimates were $V_{init} = 21.2719$ ml and $k_{init} = 0.007617$ h⁻¹.

Final parameter estimates and between-subject and between-occasion variance estimates are reported in Table G1. For the estimation of the individual parameters by conditional mode, k varied between 0.00718 and 0.00860 h^{-1}, with a shrinkage statistic of 25.5%. The estimated elimination rate constant corresponded to an ocular half-life of 3.58 days, using equation (5) from the main text to make the conversion.

	Value	Standard error (absolute)
V_{pop} (ml)	14.6	0.599
k_{pop} (h^{-1})	0.00798	0.000139
ω_k (IIV on k)	0.0474	0.0131
γ_k (IOV on k)	0.0295	0.0114

Table G1. Final population parameter estimates from the nonlinear mixed-effects model. V_{pop} (mL) and k_{pop} (h^{-1}) represent the typical population values. Inter-individual (ω_k) and inter-occasion (γ_k) variability on k were modelled using exponential random effects; reported values correspond to the estimated standard deviations of the random effects. Standard errors (absolute) were derived from the Fisher Information Matrix in Monolix.

H. Application of the rabbit-derived model to human axial length data

To contextualise the translational implications of our findings in relation to previously reported human data, we applied the rabbit-derived relationship between axial length and half-life to the clinical dataset reported by Krohne et al.[9] In a post hoc analysis, Krohne et al. investigated the relationship between axial length and aqueous humour concentrations of ranibizumab and bevacizumab (anti-VEGF therapeutics) in 41 eyes from 41 patients. Specifically, they examined the association between axial length and the relative deviation from the mean drug concentration and reported no significant correlation.

We extrapolated the linear relationship between axial length (AL) and half-life derived from the rabbit concentration-data fits (Figure 10**b**) across the axial length range reported in their study, 21.3–24.9 mm. This corresponds to predicted ocular half-lives of 5.11–6.44 days based on the linear relationship in rabbits:

$$t_{1/2} = 0.37 * \mathrm{AL} - 2.77,$$

corresponding to elimination rate constants $\lambda = 0.136$ d^{-1} to $\lambda = 0.108$ d^{-1}. For simplicity, we additionally assumed that the initial vitreous volume (V_0) scales directly with AL under a spherical-eye approximation. Using equation (3) from the main text, ranibizumab

concentrations following a 0.5 mg intravitreal injection were simulated over time across this AL range. The resulting concentration profiles are shown in Figure H1, together with the profile corresponding to the mean AL value reported by Krohne et al. (23.1 mm).

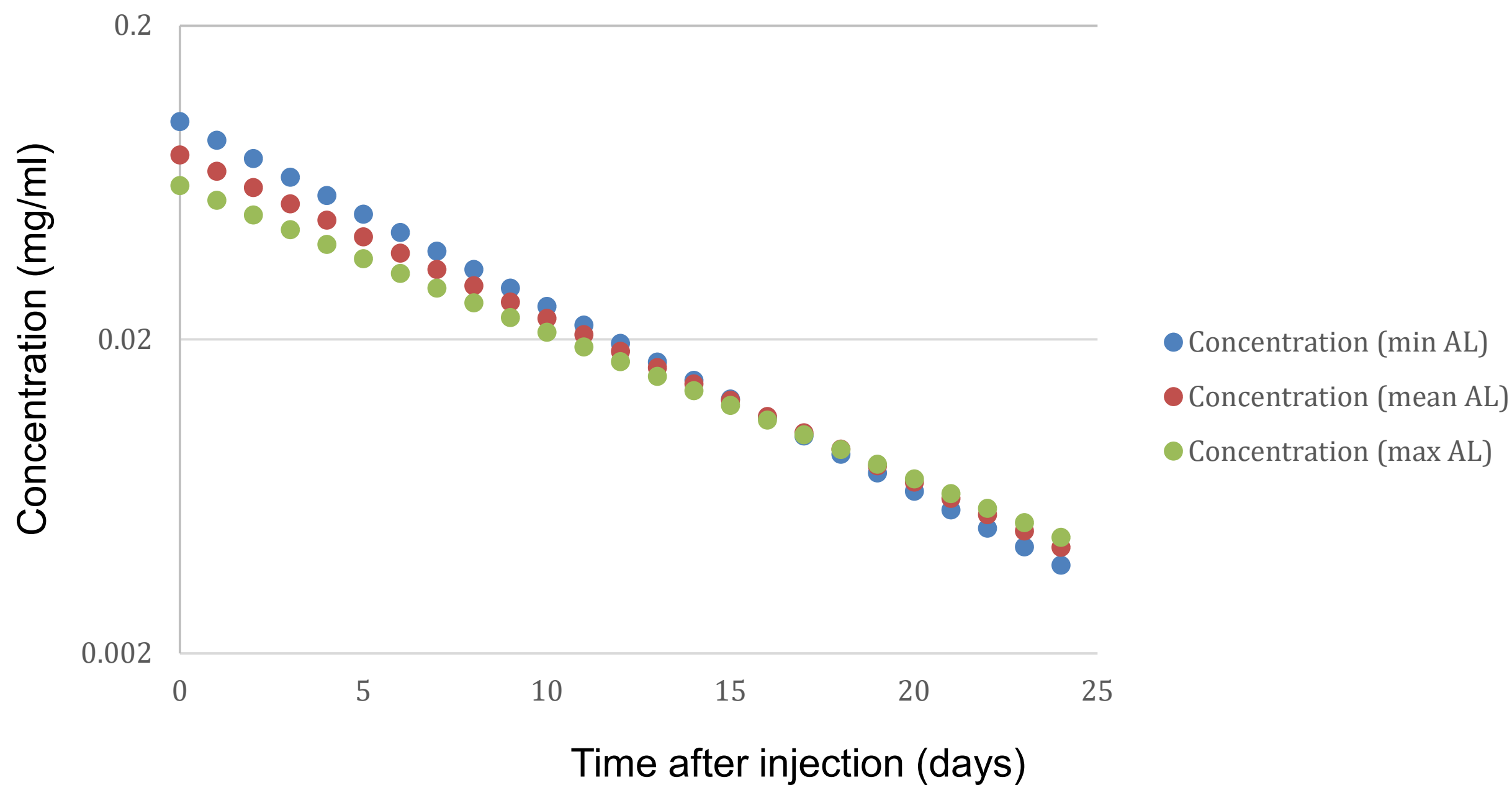

Figure H1. Modelled concentration of ranibizumab after intravitreal injection of 0.5 mg, for axial lengths varying between 21.3 mm to 24.9 mm (minimum and maximum shown in green and blue, respectively), with the mean AL of 23.1 mm shown in red.

To facilitate comparison with the analysis reported by Krohne et al., concentration deviations relative to the mean AL profile were calculated for the minimum and maximum axial lengths. The predicted deviations, evaluated at daily sampling intervals from 1 to 24 days after injection, were compared with digitised data extracted from Figure 2 of Krohne et al. and are shown in Figure H2. The 24-day limit was selected to match the methodology of Krohne et al., who excluded ranibizumab concentration measurements obtained more than 24 days after injection. These simulations suggest that the relationship between AL and ocular half-life predicted by our modelling framework would be difficult to detect using single time-point concentration measurements. This is partly because the magnitude and sign of the concentration deviation depend on the sampling time following injection, resulting in substantial variability across the observation window. In addition, the predicted effect of AL on half-life is relatively modest over the axial length range considered.

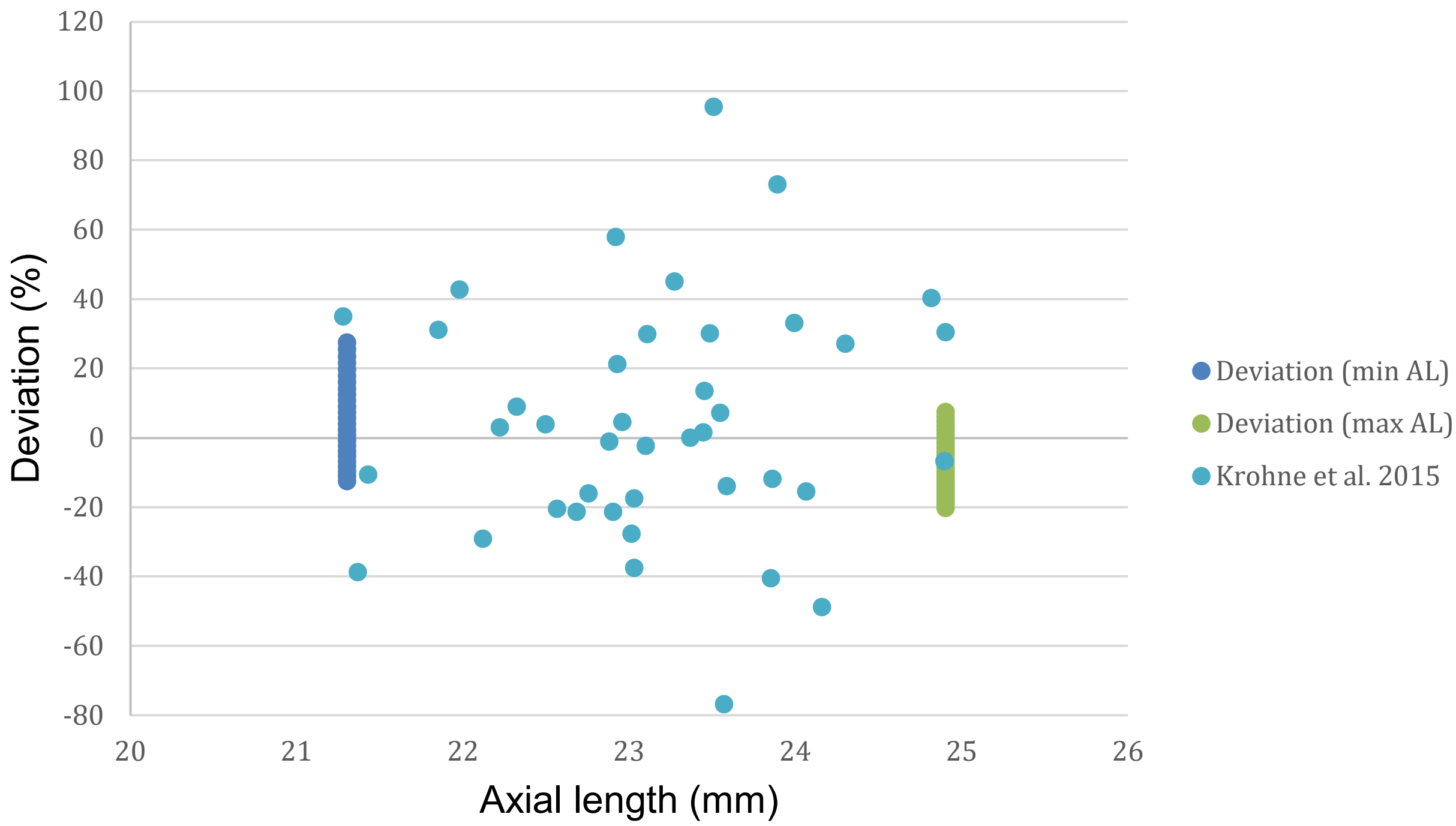

Figure H2. Deviation of drug concentration from the mean (%): light blue is ranibizumab and bevacizumab digitised data points from Krohne et al.[9], dark blue and green are the predicted deviation from our simple model described above for the minimum and maximum axial lengths, respectively.

Importantly, the limited detectability of this relationship in single time-point concentration measurements does not imply a negligible effect on clinical treatment regimens. As discussed in the main manuscript, ranibizumab is typically administered at 28-day intervals, corresponding to approximately five half-lives, such that relatively small differences in elimination half-life may accumulate over a dosing cycle.

Reference

1. Lamirande P, Gaffney EA, Gertz M, Maini PK, Crawshaw JR, Caruso A. A First-Passage Model of Intravitreal Drug Delivery and Residence Time—Influence of Ocular Geometry, Individual Variability, and Injection Location. *Invest Ophthalmol Vis Sci*. 2024;65(12):21-21.

2. White H. A heteroskedasticity-consistent covariance matrix estimator and a direct test for heteroskedasticity. *Econom J Econom Soc*. Published online 1980:817-838.

3. Kolmogorov AN. Sulla determinazione empirica di una legge di distribuzione. *G Ist Ital Attuari*. 1933;4:89-91.

4. Smirnov NV. On the estimation of the discrepancy between empirical curves of distribution for two independent samples. *Bull Math Univ Moscou.* 1939;2(2):3-14.

5. Atchison DA, Jones CE, Schmid KL, et al. Eye shape in emmetropia and myopia. *Invest Ophthalmol Vis Sci.* 2004;45(10):3380-3386.

6. Azhdam AM, Goldberg RA, Ugradar S. In vivo measurement of the human vitreous chamber volume using computed tomography imaging of 100 eyes. *Transl Vis Sci Technol*. 2020;9(1):2-2.

7. de Santana JM, Cordeiro GG, Soares DT, Costa MR, Paashaus da Costa Pinto A, Lira RP. Use of axial length to estimate the vitreous chamber volume in pseudophakic. *Graefes Arch Clin Exp Ophthalmol*. 2021;259:1471-1475.

8. Simulations Plus. Monolix. Published online 2024. doi:doi: 10.5281/zenodo.11401936

9. Krohne TU, Muether PS, Stratmann NK, et al. Influence of ocular volume and lens status on pharmacokinetics and duration of action of intravitreal vascular endothelial growth factor inhibitors. Retina. 2015;35(1):69-74.
